# Estimating death rates in complex humanitarian emergencies using the network survival method

**DOI:** 10.1093/aje/kwaf101

**Published:** 2025-05-07

**Authors:** Casey F Breen, Saeed Rahman, Christina Kay, Joeri Smits, Abraham Azar, Steve Ahuka, Dennis M Feehan

**Affiliations:** Department of Sociology, Center on Aging and Population Sciences, and Population Research Center, The University of Texas at Austin, Austin, TX, United States; Leverhulme Centre for Demographic Science, Department of Sociology and Nuffield College, University of Oxford, Oxford, United Kingdom; IMPACT Initiatives, Geneva, Switzerland; IMPACT Initiatives, Goma, Democratic Republic of the Congo; IMPACT Initiatives, Geneva, Switzerland; Department of Economics, Tufts University, Medford, MA, United States; IMPACT Initiatives, Geneva, Switzerland; School of Public Health, University of Kinshasa, Kinshasa, Democratic Republic of the Congo; Department of Demography, University of California, Berkeley, CA, United States

**Keywords:** humanitarian emergencies, mortality estimation, network survival method

## Abstract

Reliable estimates of death rates in complex humanitarian emergencies are critical for assessing the severity of a crisis and for effectively allocating resources. However, in many humanitarian settings, logistical and security concerns make conventional methods for estimating death rates infeasible. We develop and test a new method for estimating death rates in humanitarian emergencies using reports of deaths in survey respondents’ social networks. To test our method, we collected original data in Tanganyika Province of the Democratic Republic of the Congo (*n* = 5311), a setting where reliable estimates of crude death rates (CDR) are in high demand. Qualitative fieldwork suggested testing 2 different types of personal networks as the basis for CDR estimates: deaths among immediate neighbors and deaths among kin. We compare our network-based estimates (0.44 deaths per 10 000 person-days) against a standard retrospective household mortality survey, which estimated a CDR nearly twice as high (0.81 deaths per 10 000 person-days). Given that both methods are equally plausible, our findings highlight the need for further validation and development of both methods.

## Introduction

Reliable estimates of death rates are essential for addressing complex humanitarian emergencies. These estimates are crucial for crisis assessment, resource allocation, preserving the historical record of tragedies, and supporting advocacy.^1^^-^^3^ Recent estimates of mortality in humanitarian emergencies have guided effective responses to armed conflicts,^4^^-^^6^ famine,^7^ and war crimes.^8^

The most reliable way to learn about death rates is generally through data from a high-quality civil registration and vital statistics system (CRVS). However, during complex humanitarian emergencies, this is often not feasible. In some settings, high-quality CRVS systems may not exist; in others, the system may deteriorate over the course of the emergency.^9^ For instance, at the time of the 2010 earthquake in Haiti, there was no high-quality CRVS system,^10^ and even if there had been, the earthquake caused a near-total collapse of civic infrastructure and processes.^11^ Therefore, alternative methods of estimating death rates are needed.

Existing methods fall into 3 broad classes: First, retrospective household mortality surveys are a widely used approach for estimating death rates.^12^^-^^16^ These surveys typically involve asking a probability sample of households about vital events and household composition during a recall period.^17^ Household surveys are time-consuming and costly and, even when well executed, can be prone to various errors leading to underestimation or overestimation of death rates.^18^^-^^20^ For example, Jarrett et al.^21^ conducted a careful validation exercise, comparing deaths reported in a surveillance system and a retrospective household survey. They found that more than half of the deaths reported in the survey were either outside the recall window, occurred in a different household, or were fabricated. In practical terms, in humanitarian emergencies, obtaining a high-quality probability sample is often challenging or impossible. For instance, data collection was paused for 3 weeks in response to major security concerns, including the attack and burning of a data collection office in a 2004 mortality household survey in the Democratic Republic of the Congo.^22^ Therefore, household mortality surveys are generally not a feasible strategy for estimating real-time mortality in humanitarian emergencies.^23^

Second, prospective demographic surveillance systems can be established for monitoring deaths.^24^ In a prospective demographic surveillance system, trained enumerators visit homes and administer surveys, collecting data on deaths, births, and migration for prespecified time intervals (eg, weekly, monthly). Ideally, this approach would provide real-time death rates, but in practice, updates occur only when new deaths are reported, which may happen only a few times per year. Additionally, properly enumerating the population denominator can take several months. Moreover, such surveillance systems are expensive, difficult to maintain, and often deteriorate in complex humanitarian emergencies.^2^^,^^25^^,^^26^

Third, key informant reporting involves selecting key informants to report on deaths within a predefined community, such as a village or neighborhood.^27^ Using capture-recapture methods, these data can be combined with lists of deaths from other sources to estimate death rates.^28^^,^^29^ This approach is more cost-efficient than surveillance systems or retrospective surveys, but a validation study conducted in 4 separate study sites found this approach undercounts deaths among children younger than 5 years.^27^ In certain types of humanitarian emergencies, selecting appropriate key informants may be challenging, and informants may struggle to accurately report on displaced populations. Future empirical work will be useful in furthering our understanding of the settings in which this method can be successfully applied.

Each of these methods is important but has limitations that are exacerbated in humanitarian emergencies. There remains an urgent need for specialized methods to estimate timely death rates in humanitarian emergencies.^23^ In this study, we adapt a method called network survival to the challenge of estimating death rates during a complex humanitarian emergency in which operational constraints prevent direct access to populations. The network survival method was originally developed to estimate national death rates.^30^ Our study builds on this earlier work by introducing several key methodological innovations, including: 1) using a nonprobability sampling approach that allows remote data collection without an on-the-ground presence; 2) using a short retrospective window to facilitate high-frequency death estimates; 3) incorporating qualitative work to inform the specific choice of networks for reporting; and 4) refining methods for blending 2 death rate estimates.

## Study design and data collection

### Study site

**Figure 1:**
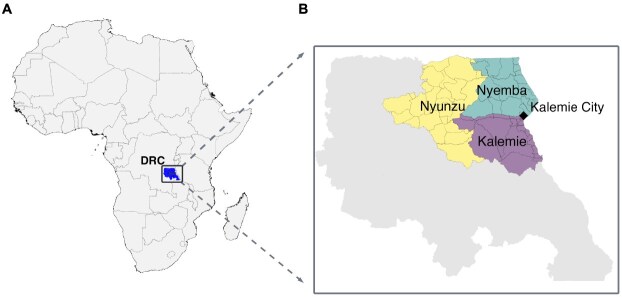
A) Map of Africa with the Democratic Republic of the Congo’s (DRC) Tanganyika Province highlighted. B) Inset of Tanganyika Province, with the 3 focal health zones of this study highlighted. The quota survey respondents were all sampled in Kalemie City (black diamond). The household survey respondents were sampled in their respective health zones.

To empirically test our new method, we needed a setting that satisfied 2 criteria: 1) it should have characteristics similar to other places where humanitarian emergencies have emerged in the past; and 2) it should be possible to obtain a probability sample that could produce a set of estimates using a standard retrospective household survey. We chose 3 health zones in the Tanganyika Province of the Democratic Republic of the Congo: Kalemie, Nyemba, and Nyunzu (Figure 1).

These health zones border one another in the easternmost part of the country, which is characterized by high death rates and historical insecurity problems that have resulted in humanitarian emergencies in the past.^31^^,^^32^ This region is an example of the kind of setting where humanitarian emergencies may emerge and methods for estimating death rates are critically needed. Furthermore, in collaboration with our partner organization, IMPACT Initiatives, we determined it would be possible to obtain a probability sample of households to produce estimates from a standard probabilistic household survey.

### Design and data collection

**Figure 2:**
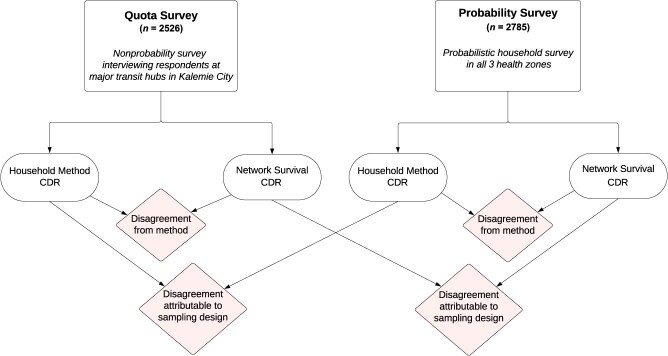
Illustration of study design and comparisons.

Our design called for 2 separate data collection projects that produced several different estimates of the crude death rate (CDR) (Figure 2). The first data collection project used the network survival approach to produce CDR estimates from a sample that could realistically be obtained during a humanitarian emergency. In such emergencies, a conventional probability survey would likely be infeasible due to security and logistical challenges. Instead, we collected a nonprobability quota sample designed to imitate a setting where operational constraints prevent humanitarian actors from reaching insecure areas, but populations may be moving back and forth to access services and markets or evacuating an insecure area (“quota sample”). The network survival method uses survey questions to collect information about deaths and exposure among respondents’ personal networks (eg, kin, neighbors); thus, it is possible to learn about people and places that cannot directly be reached by the study team. As we describe, data collected from the quota sample allowed us to produce several different CDR estimates based on the network reports. We also asked quota-sample respondents retrospective questions about deaths in their households.

The second data collection project aimed to produce a set of CDR estimates using the standard approach: a retrospective, probabilistic household survey. We used a probability sampling design to obtain a sample of households in the study area (“probability sample”). Enumerators visited these households in person and interviewed respondents using a standard survey instrument, which included questions about deaths among household members. Respondents were also asked the same network reporting questions used in the quota-sample survey. Retrospective probabilistic household surveys are known to have some flaws,^18^^-^^20^ and they are generally not feasible in a humanitarian emergency. However, there is no perfect way to estimate mortality from a survey, and retrospective household surveys have been widely adopted by governments and nongovernmental organizations (NGOs).^33^^,^^34^ In our study, the probability-based household estimates served 2 purposes: 1) they allowed us to separate out the effect of nonprobability sampling and the effect of reporting about network members on the network survival estimates; and 2) they allowed us to have a set of estimates produced by a standard (if imperfect) method that can be used to contextualize and compare estimates from the new method.

Our primary comparisons are outlined in Figure 2. From both the quota sample and probability sample, we produced separate estimates of the CDR using the household method and the network survival method. Within each sample, we compared the household and network estimates to understand the difference attributable to methods. Within each method, we compared the probability and quota estimates to understand the difference attributable to sampling strategy.

The quota survey targeted adults aged 18 years or older using a nonprobability, quota-based sampling strategy to intercept people coming into Kalemie City from all 3 health zones. Specifically, trained survey enumerators sampled respondents at transit hubs and service sites, including ports, markets, taxi stands, foot paths, and health clinics, in Kalemie City. The quotas were established based on sex and health areas, the geographic units below health zones. A total of 2526 interviews were conducted in Kalemie City between March 1 and June 29, 2023. Respondents answered demographic and socioeconomic questions before reporting deaths within their kin and neighbor networks. Respondents were asked to report about deaths that occurred between January 1, 2023, and their interview date.

An important limitation of our quota sample is that it only accounted for sex and geography; it did not account for other dimensions of selection, such as age or socioeconomic status. In general, there is a trade-off between representativeness and feasibility when implementing quotas: more complex quotas improve representativeness but can be challenging to implement, whereas simpler quotas may not be representative of the general population. Despite these limitations, quota surveys have been effective across a range of public health settings where probability sampling was infeasible due to logistical or financial constraints, including studies on conflict-affected populations in South Sudan,^35^ interpersonal contact during the COVID-19 pandemic,^36^ and mortality estimation in Ebola-affected regions.^37^

The survey used a randomized order for the kin and neighbor modules, and questions were placed in subcategories to reduce cognitive load. To help reach more remote areas of the Nyunzu Health Zone, we established a secondary sampling site in Nyunzu Town. However, the sole enumerator in Nyunzu Town had limited direct supervision, leading to some potential data quality concerns. Because this enumerator only conducted 30% of overall interviews for respondents living in Nyunzu, we dropped these data from our main analysis. A robustness check demonstrated no statistically significant differences in our estimates if these data were or were not included in our analysis (see Appendix S4 for details).

The probability survey was fielded directly after the quota survey, from July 21, 2023, to September 1, 2023. The probability survey was conducted in person at 2785 households in randomly sampled clusters across all 3 health zones. Respondents reported on deaths since January 1, 2023, the same reference date as the network survey. The probability survey included the complete network survival module, allowing us to produce both a standard household estimate and a network survival estimate of the CDR. The full survey instrument is available in Appendix S6. 

### Quantity of interest

Our primary quantity of interest was the CDR, expressed in deaths per 10 000 people per day. These are the units typically used in complex humanitarian settings to estimate CDRs.^4^^,^^38^^,^^39^ To convert to the standard units used in demography, deaths per 1000 people per year, multiply the CDR by 36.5 (for details, see Appendix S2.3). Mathematically, the CDR *M* is given by $M=\frac{D}{N}\times 10\ 000$, where *D* is the number of deaths that occurred in a given period and *N* is the total person-days of exposure. We estimate the pooled CDR across all 3 health zones from January 1, 2023, to June 29, 2023.

### Formative fieldwork

To help inform the design of our study, we conducted 8 focus groups and 25 open-ended interviews in the study setting. The primary goal of this formative research was to identify the specific personal network(s) for respondents to report on: networks that were large enough for us to learn much from each interview but small enough to accurately recall and report death.^40^ The formative research also informed other study parameters, including the recall period length, the method for estimating network size, and the selection of transit hubs and service sites (eg, ports, taxi stands, markets) for sampling respondents. Our qualitative fieldwork suggested using 2 different types of personal networks as the basis for death rate estimates shown in Table 1: deaths among immediate neighbors and deaths among kin.

**Table 1:** Household, kin, and neighbor network subgroups.

**Network tie**	**Group**
Household	Respondent’s household
Neighbor	1st Closest neighbor household
Neighbor	2nd Closest neighbor household
Neighbor	3rd Closest neighbor household
Neighbor	4th Closest neighbor household
Neighbor	5th Closest neighbor household
Kin	Respondent’s grandchildren
Kin	Respondent’s children
Kin	Respondent’s siblings
Kin	Respondent’s cousins
Kin	Respondent’s aunts/uncles
Kin	Respondent’s parents
Kin	Respondent’s grandparents

One potential issue in mortality estimation studies is recall bias, which occurs when respondents systematically forget or otherwise misreport past events, leading to inaccuracies in reported deaths. In the context of mortality estimation, this can result in underreporting of deaths, particularly when respondents struggle to recall exact dates or do not report deaths that occurred further in the past. To mitigate recall bias, we selected a significant and locally memorable reference event—New Year’s Day, January 1, 2023—as the starting point for the recall period. Research has shown that anchoring recall to well-known events, such as Ramadan or the death of a political leader, improves accuracy in reporting deaths.^39^ Our qualitative research revealed that New Year’s Day was highly salient in this setting and helped respondents better remember whether a death occurred in the recall period. We also selected a relatively short recall window, which, at maximum, was 8 months long, to further minimize recall bias.

## Estimation

### Network survival method

Building on the broader network reporting literature for studying hard-to-reach populations,^41^^-^^44^ the network survival method can be thought of as a generalization of the sibling method^45^^-^^49^ and the network scale-up method.^41^ The network survival method has generated estimates that closely align with those produced by international health organizations in a similar setting in Rwanda, using a probability survey.^30^ Furthermore, in Brazil, more than 25 000 respondents were probabilistically sampled across 27 different cities,^50^ and the network method estimates were benchmarked against the gold standard vital statistics collected by the Brazilian government. The estimates aligned closely with the ground truth estimates from vital statistics and were 15% more accurate at modest sample sizes (*n*  $\approx$ 1000) than the standard sibling method.

The core idea of the network survival method is to ask respondents to report about deaths occurring within their personal networks. Specifically, the network method asks a survey respondent to answer a series of questions that can be used to determine 1) how many people are in the respondent’s personal network; and 2) how many people in the respondent’s personal networks died in a given period. These network reports are then combined to estimate a death rate:


(1) \begin{equation*} \hat{M}=\left(\frac{\hat{D}}{\hat{N}}\right)\times \mathrm{10,000}=\left(\frac{{\hat{y}}_{F,D}}{{\hat{y}}_{F,N}}\right)\times \mathrm{10,000}, \end{equation*}

where $\hat{M}$ is an estimator for the CDR; $\hat{D}$ is an estimator for the number of deaths in the population; $\hat{N}$ is an estimator for the amount of exposure; *F* is the frame population (ie, the universe of people eligible to respond to the survey); ${\hat{y}}_{F,D}$ is an estimate of the total number of reported deaths among personal network members over the reference period; and ${\hat{y}}_{F,N}$ is an estimate of the total amount of exposure among personal network members reported over the reference period.

### Network survival estimator

To use this estimator in our study, we had to specify estimators for ${y}_{F,D}$ and ${y}_{F,N}$. The expression for estimating reported deaths, ${y}_{F,D}$, can be written as


(2) \begin{equation*} {\hat{y}}_{F,D}=\sum \limits_{i\in s}{w}_i\ {y}_{i,D}, \end{equation*}

where *s* is the sample; ${w}_i$ is a weight for respondent $i\in s$; and ${y}_{i,D}$ is the number of deaths among personal network members reported by respondent *i*. The expression for estimating reported exposure, ${\hat{y}}_{F,N}$, can be written as


(3) \begin{equation*} {\hat{y}}_{F,N}=\sum \limits_{i\in s}{w}_i\ {d}_i\ {E}_i, \end{equation*}

where ${d}_i$ is the reported number of people in *i*’s personal network and ${E}_i$ is the number of days of exposure respondent *i* reported about their personal network. The product of ${d}_i$ and ${E}_i$ estimates the total amount of exposure reported by respondent *i* in person-days.

Putting eqns. (2) and (3) together with eqn. (1), we have the estimator we used in our study:


(4) \begin{align*} \hat{M}=\left(\frac{\sum \limits_{i\in s}{w}_i\ {y}_{i,D}}{\sum \limits_{i\in s}{w}_i\ {d}_i\ {E}_i}\right)\times \mathrm{10,000}. \end{align*}

Eqn. (4) is convenient because it expresses the estimator in terms of respondent-specific weights, ${w}_i$.

### Producing estimates from the quota sample

**Figure 3:**
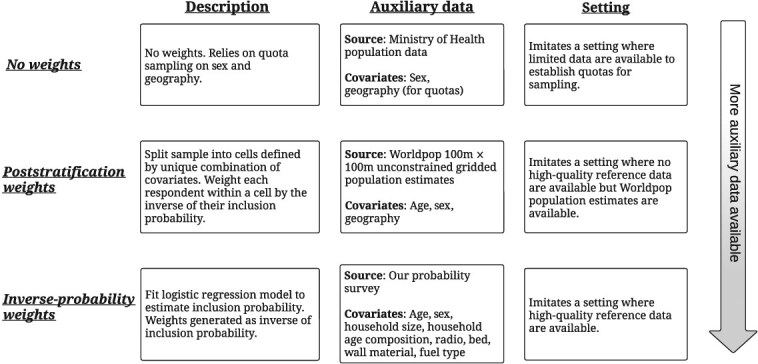
Overview of different weighting strategies. We developed weighting strategies intended to mimic the availability of increasingly rich external data.

Our design called for quotas by sex and health area, the geographic units below health zones. These allowed us to closely match the overall target population’s sex and geographic distributions. However, the quota did not account for selection with respect to socioeconomic status, age, or other characteristics. Quota-sample respondents were wealthier, and the youngest and oldest age segments were underrepresented compared with the general population (see Figure S1 for more details). To address this, we developed weighting strategies intended to mimic the availability of increasingly rich external data (Figure 3): unweighted estimates relying solely on our quota sample, estimates using WorldPop gridded population data for poststratification weights, and estimates with inverse probability weights based on respondents’ age, sex, household size, household age composition, and ownership of assets that correlate with household wealth. We constructed inverse probability weights using logistic regression to model inclusion probability in the quota sample based on a pooled quota and probability sample.^51^

Inverse probability weighting (IPW) is the preferred weighting approach for this method when sufficient auxiliary data are available, because it can more easily adjust for a broad range of characteristics. However, IPW relies on the availability of high-quality auxiliary data to weight against (eg, a reliable census or probability survey), and measurement errors in auxiliary data may bias the adjusted estimates. Furthermore, IPW can lead to unstable weights when probabilities of selection are very small, resulting in high variance. Finally, all weighting strategies can only account for the measured dimensions of selection and cannot address bias from unmeasured factors. For more details on weighting, see Appendix S2.6 and Table S2. 

To assess sampling uncertainty, we constructed 10 000 bootstrap resamples. Each resample was drawn with respect to sex, health zone, and survey month, mirroring our original quota sampling design. From each bootstrap resample, we calculated a point estimate of the CDR. Using the 2.5th and 97.5th percentiles of these CDR estimates, we constructed a 95% uncertainty interval. This approach quantifies the uncertainty in our estimates due to the randomness of the sampling process.

We produced separate estimates using reports about neighbor and kin networks. In addition, we use a blended estimator to produce a combined estimate based on both the kin and the neighbor network reports.^40^ Specifically, the blended estimate is based on averaging together the estimate from each network in a principled way. Suppose we have 2 estimators for *N*, ${\hat{N}}^A$ and ${\hat{N}}^B$. We define the blended estimate with pooling weight $\theta$ as


(5) \begin{equation*} \underset{\mathrm{Blended}\ \mathrm{Estimator}}{\underbrace{\hat{N}}}=\underset{\mathrm{Weighted}\ \mathrm{Estimator}\ \mathrm{A}}{\underbrace{\theta{\hat{N}}^A}}+\underset{\mathrm{Weighted}\ \mathrm{Estimator}\ \mathrm{B}}{\underbrace{\mathrm{\big(1}-\theta \big){\hat{N}}^B}} \end{equation*}

where $\theta$ is a weight $\in \mathrm{\left[0,1\right]}$. The advantage of this blended approach is that we expect it to produce smaller mean squared error than either kin or neighbor estimate alone, because the estimate is based on more information. But this comes at the cost of additional assumptions; see Appendix S2.7 and Feehan et al.^40^ for a detailed discussion and derivation of the optimal weight.

### Producing estimates from the probability sample

We produced CDR estimates from the probability sample using 2 methods: the standard household method and the network survival method. For the standard household method, we calculated person-time observed for each individual based on relevant dates within the recall period, such as dates of birth, death, joining the household, or leaving the household. We then calculated the CDR by dividing the number of deaths by the total person-time observed and re-scaling to express as deaths per 10 000 person-days. To generate network survival estimates from our probability sample, we applied the same estimator used for the quota sample. However, we did not use survey weights, because we considered the probability sample self-weighting. To make the probability survey estimates directly temporally comparable to the quota sample estimates, we restricted the probability sample to deaths and exposure reported during the same recall period as the quota sample (January 1, 2023, to June 29, 2023).

## Results

**Figure 4:**
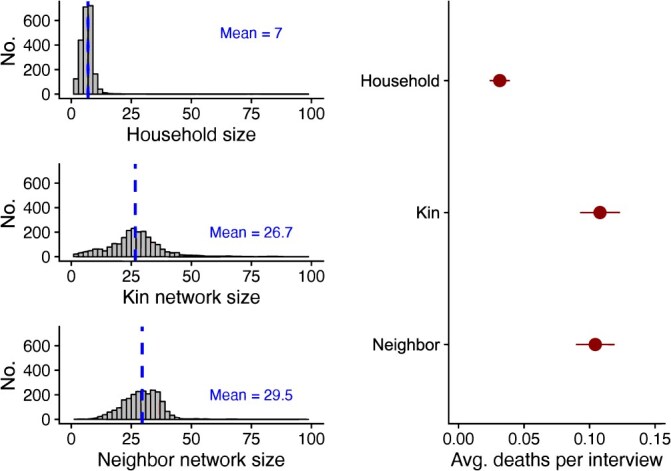
Network size and average (Avg.) deaths per interview from quota sample. Uncertainty bars show 95% CIs.

First, we analyze our estimates from the quota sample. Figure 4 shows the distribution of household and network sizes. The average household size was 7 people. In comparison, the average kin network size was 26.7 people, and the average neighbor network size was 29.5 people. Correspondingly, respondents reported many times more deaths in their neighbor and kin networks than in their own household.

**Figure 5:**
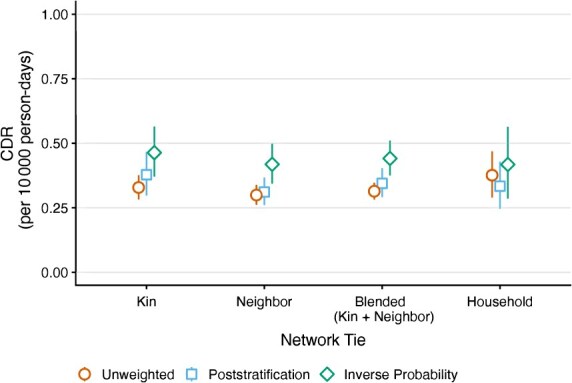
Network survival method estimates of the crude death rate (CDR) from the quota sample under 3 different weighting schemes. The CDR is expressed in units of deaths per 10 000 person-days. Uncertainty bars show 95% CIs.

Figure 5 presents 3 sets of CDR estimates, based on kin reports, neighbor reports, and a blended combination of the 2. For each, we calculate 3 estimates: unweighted, poststratified (adjusted for sex, age, and geography), and inverse probability weighted (incorporating all available sociodemographic information). This allowed us to assess how weighting adjustments influenced our estimates.

To incorporate respondent-specific weights, we weighted death reports and exposure contributed by each individual, as described in eqn. (4). Poststratification increased estimates slightly, except for household-based estimates, which declined modestly. In contrast, IPW raised estimates by approximately 40% for both kin and neighbor networks. Despite some variation, kin and neighbor estimates remained consistent across weighting strategies. Household estimates were noisier but generally aligned with network-based estimates.

Next, we compared estimates from our quota sample with estimates from our probability sample. For ease of comparison, we focused on what we would expect to be our best network estimate from our quota sample: our blended estimates with inverse probability weights. The blended network IPW estimate was 0.44 (95% CI, 0.38-0.51), closely aligning with the kin (0.46; 95% CI, 0.37-0.56) and neighbor (0.42; 95% CI, 0.34-0.50) IPW estimates. This blended estimate combines information from neighbor and kin reports and weights to account for selection into our quota sample.

**Figure 6:**
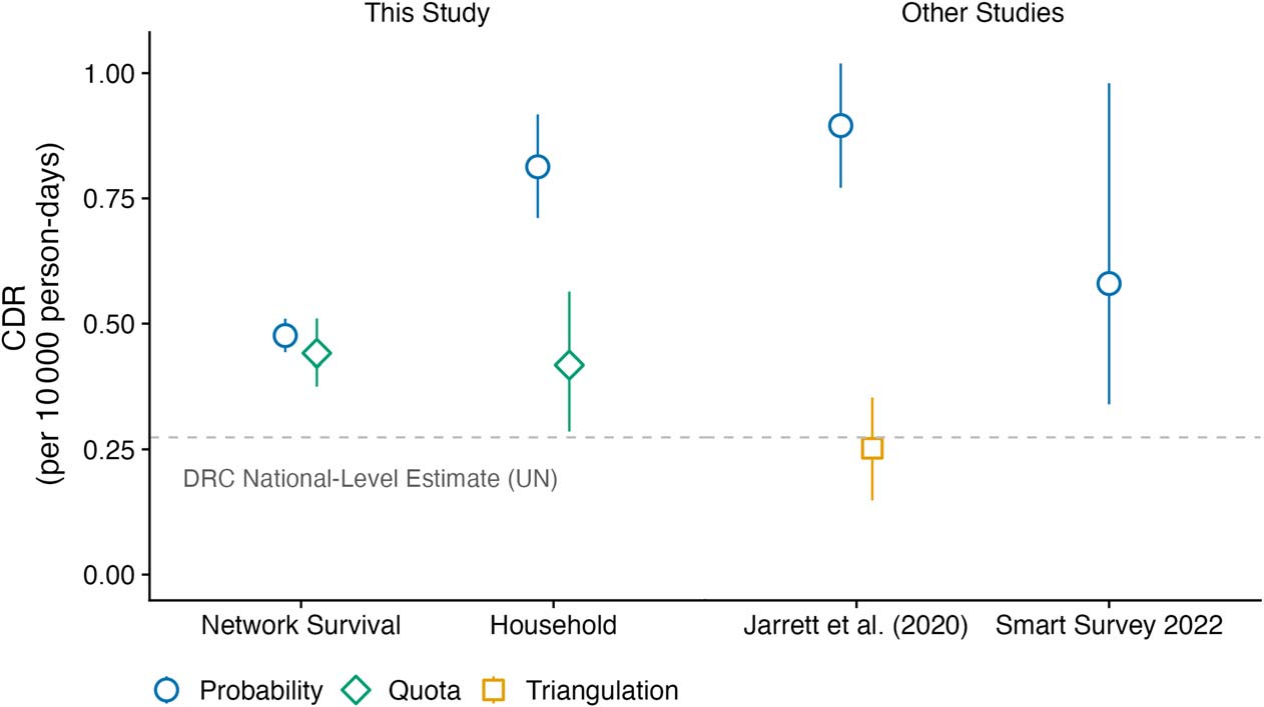
Comparison with crude death rate (CDR) estimates from other studies. The quota sample estimates were weighted using inverse probability weights; the network survival estimates were blended estimates from both kin and neighbor networks. The Jarrett et al.^21^ study was conducted in the Fizi province, Democratic Republic of Congo (DRC) in 2011. The 2022 Standardized Monitoring and Assessment of Relief and Transitions (SMART) survey was conducted in the Kalemie Health Zone in November 2022. Uncertainty bars show 95% CIs. Dashed gray horizontal line shows DRC national-level estimate from the United Nations.

Figure 6 presents the full set of comparisons between both arms of our study and external estimates. The blended network CDR estimate from the quota sample of 0.44 (95% CI, 0.38-0.51) aligned closely with the blended network estimate from the probability sample of 0.48 (95% CI, 0.44-0.51). Additionally, within our quota sample, CDR estimates based on household reports were consistent with both network estimates. However, the probability sample household estimate was substantially larger than both our quota sample household estimate and all network estimates.

To help contextualize this disagreement, we made several comparisons with other external studies, noting that these external estimates were neither perfectly temporally nor geographically aligned with ours. Our first comparison was to a Standardized Monitoring and Assessment of Relief and Transitions (SMART) survey conducted in November 2022 in the Kalemie Health Zone.^52^ This study asked respondents to report on deaths after August 1 and before the November interview date, an observation window approximately 6 months before our observation window. This survey found a CDR of 0.58 (95% CI, 0.34-0.98), slightly higher than the quota-blended IPW estimate (0.44; 95% CI, 0.38-0.51).

Next, we compared our estimates with those reported by Jarrett et al.^21^ In their study, Jarrett et al. collected data in the Fizi Health Zone in 2011 and 2012. The Fizi Health Zone borders our study area to the north (see Figure S2 and Appendix S2.8). Despite these estimates being over 10 years old and from a neighboring health zone with potentially differing contexts of conflicts, disease outbreaks, and population dynamics, they still provided valuable insights into the reliability of standard household-based CDR estimates in these settings. A standard, probabilistic household survey found a CDR of approximately 0.9 (95% CI, 0.77-1.02). To understand how accurate the probabilistic household survey was, Jarrett et al.^21^ conducted a separate surveillance of households and then re-interviewed all household respondents to reconcile any discrepancies between the surveillance and household survey. This careful reconciliation process found only 28% of deaths reported in the household study had legitimately occurred in the study period; of the 72% of erroneous death reports, these “deaths” were either outside the recall period (32%), not within the household (48%), or false reports (20%). After this reconciliation process, the authors estimated a CDR of 0.25 (95% CI, 0.15-0.35) and hypothesized that strategic overreporting was responsible. The Jarrett et al.^21^ study adds to a growing body of literature highlighting that household surveys—even when well executed and administered—may produce biased estimates of mortality.^18^^-^^21^ These studies reinforce the need to be cautious when interpreting our own household-based estimates, which may potentially be affected by similar biases, including strategic overreporting.

We performed a series of validation checks to confirm the quality of our survey responses and network reports (see Appendix S3 for full details). First, to test for respondent fatigue during interviews, we compared responses based on the randomized order of our network modules. Regardless of the survey order, respondents reported nearly identical average household sizes and average numbers of deaths, indicating consistent and reliable responses (Figure S6). Second, we performed internal validity checks for network reports, focusing on relationships we expected to be reciprocal (Figure S4). The results showed no significant deviations from reciprocity, further confirming the reliability of the network reports. Finally, we compared the age composition of the quota sample (ie, household, kin, and neighbor networks) with the age composition of the probability sample and found high overall agreement (Figure S5).

## Discussion

In this study, we introduce a new method for estimating death rates by adapting the network survival method to nonprobability settings, as demanded by the unique constraints of humanitarian emergencies. We conducted formative fieldwork to help us pick which personal networks to ask respondents to report on, recall period length, and other important design parameters. We assessed the performance of this method in a realistic setting and conducted a probabilistic household survey for comparison. Although the limitations of household-based mortality surveys are well documented^18^^-^^21^ they are widely used, and so we see them as a useful comparator, if not a gold standard. This comparison helps us better understand the plausibility of both sets of estimates and different potential sources of error relative to the ground truth. Taken together with external estimates, our results highlight a large amount of uncertainty about the true underlying CDR in our focal health zones. Despite the lack of a reliable ground truth to benchmark our CDR estimates against, our study had several key findings.

Our quota sample taken at transit hubs and service sites in Kalemie City was positively selected with respect to socioeconomic status compared with the broader population. This was expected, because our quotas for the sample were only based on sex and geographic region (health area) and did not address selection into the sample with respect to age or socioeconomic status. In our quota samples, our weighted estimates were substantially higher than our unweighted estimates. This suggests that adjusting for socioeconomic selection into the nonprobability sample is crucial for producing accurate estimates and indicates that, as expected, people with lower socioeconomic status in this setting had neighbors and kin with higher incidence of death. After reweighting to adjust for selection, our network estimates from the quota sample aligned closely with our network estimates from our probability sample. Despite the major differences in sample design, the reweighted quota and probability samples produced nearly identical network estimates, demonstrating the effectiveness of the reweighting approach in this setting. Furthermore, both network estimates were consistent with our estimated household CDR from the quota sample. However, the CDR estimate from the probability sample household reports was substantially higher than any other estimate.

This lack of agreement is surprising. Although our study cannot speak definitively to this discrepancy, we can speculate on possible explanations. Given the high level of NGO activity in this area, respondents in the probability sample may have been motivated to answer in a way that would maximize their chances of receiving aid, similar to the “strategic misreporting” hypothesized by Jarrett et al.^21^ This incentive would be stronger in the probability sample, where enumerators visited respondents’ households and could potentially return to deliver aid. In contrast, respondents in the quota sample likely had lower expectations of receiving aid, because they generally lived far from Kalemie City and did not provide specific addresses or locations for follow-up. Our study included a verbal autopsy for reported household deaths, asking detailed questions about causes, which may have reduced the likelihood of fabricated deaths, but not false reports outside the recall window or household. There may also have been a *memorial effect*, whereby the emotional salience of household members who passed away recently but prior to the observation window may result in overreporting. This may have been stronger in the probability sample in which people were interviewed in households, because the environment itself may remind respondents of deceased household members, making the emotional salience stronger.

On the other hand, it is also possible that the network survival method underestimated the true CDR. Respondents may have forgotten about deaths or been unaware of deaths in their extended networks. The quota and probability samples also had slightly different recall periods. Our qualitative research, however, suggested these factors are unlikely to produce errors big enough to explain the difference between the household and network estimates: respondents to our qualitative study reported that deaths were very salient, and they perceived themselves to be highly aware of deaths in their kin and neighbor networks. Our validity checks also found no cause for concern about data quality, though we cannot definitively rule out undiagnosed problems with the network reports.

The comparison with external estimates offered additional insights. The most directly comparable study, conducted in the Kalemie Health Zone approximately 6 months before our study, produced estimates that aligned with the network survival estimates.^52^ Another study, conducted 12 years earlier in a neighboring health zone,^21^ used a prospective mortality surveillance system to evaluate the accuracy of deaths reported on a probability-based household mortality survey similar to our household survey. The results revealed significant overreporting of deaths on the household survey. The authors hypothesized that the large presence of local and international NGOs may have led respondents to strategically make false reports about deaths in hope of receiving aid.^21^ Similar overreporting may help explain the discrepant household CDR estimate from the probability sample (see Appendix S2.9 for further discussion).

There are several important next steps for research that broadly fall into 2 key areas: additional validation efforts and methodological advancements. In terms of validation efforts, we hope this study motivates more empirical work to validate and assess the performance of both the standard household survey method and the network survival method in conflict settings. An ideal validation study would take place in a setting where high-quality, gold standard death estimates can be obtained, such as a demographic surveillance site. This study design would allow for a systematic comparison of conventional household retrospective mortality surveys and the network survival method benchmarked against the surveillance-based estimates. An independent reconciliation of any reported discrepancies could be conducted to investigate inconsistencies, helping to determine the extent of strategic overreporting, missed reporting of true deaths (false negatives), and recall bias. Such a study would provide helpful evaluation of both the standard household and network survival approaches.

From a methodological standpoint, future work could consider alternative model-based approaches to adjust for nonprobability sampling. We investigated several different weighting strategies, but future work could explicitly model mortality for subgroups and incorporate upweighting, similar to multilevel regression with poststratification.^53^ Additionally, although CDRs are a standard metric for measuring mortality in humanitarian emergencies, they depend on the overall age distribution of the population, limiting cross-context comparisons. Network-based methods could be extended to estimate age-specific death rates (requiring the collection of more detailed information on the ages of all network members) that explicitly account for differential age structure across network ties. This is particularly important because, although the kin, neighbor, and household networks’ age composition in crude age categories largely aligned in our study (Figure S5), there were subtle age composition differences across networks. Finally, in our study, we analyzed all data after data collection was completed. Respondents reported deaths occurring within an average recall period of approximately 6 months. Studies could explore the feasibility of shorter recall windows and implement a streamlined pipeline to generate estimates on a more regular basis.

The method introduced in this article addresses a long-standing call for the development of new tools to estimate mortality in humanitarian emergencies.^23^ We combined the network survival method with a quota sampling approach. This design could be deployed remotely in settings where operational constraints prevent humanitarian groups from reaching insecure areas, meaning it could potentially be applied to estimate death rates in a wide range of humanitarian emergencies. For example, a research team could establish a checkpoint outside of an ongoing humanitarian emergency. At this checkpoint, they could collect a quota sample, with quotas established based on sex, geographic region (based on when the emergency started), and other relevant characteristics. The survey instrument would collect information on deaths among immediate neighbors and deaths among kin, or some other network informed by qualitative research. With a sufficiently large sample size, CDR estimates could be generated monthly. The resulting CDR estimates could help track deaths over time, guide aid distribution, and support advocacy efforts for stronger interventions.

## Supplementary Material

Web_Material_kwaf101

## Data Availability

All data and replication code are available from https://doi.org/10.17605/OSF.IO/Y6F4Q.
