# Supplementary material for: Estimating death rates in complex humanitarian emergencies using the network survival method
Source: Am J Epidemiol. 2025 May 7;195(1):49–59. doi: 10.1093/aje/kwaf101 (PMC12780780; doi:10.1093/aje/kwaf101)
Supplement: Web_Material_kwaf101 [file web_material_kwaf101.pdf]

Supplementary Materials

Estimating death rates in complex humanitarian emergencies using the network survival method

Casey F. Breen Saeed Rahman Christina Kay Joeri Smits
Abraham Azar Steve Ahuka Dennis M. Feehan

October 9, 2025

Table of Contents

Figures S1–S8
Appendix S1–S6
Tables S1–S3

S1 Formative fieldwork

S1.1 Fieldwork Overview

The network survival method is highly flexible in that it can be used to produce estimated CDRs based on deaths reported in many different kinds of personal networks—friends, co-workers, kin, acquaintances, neighbors, etc. [1]. This flexibility can be advantageous because it means that researchers can adapt the method to different settings and study goals. But it also means that care must be taken in choosing which network to use as the basis of mortality estimates.

To help inform the design of our study, we conducted formative research in the study setting. The main goal of the formative research was to help us pick the specific personal network(s) to ask respondents to report on. However, the formative field work also helped inform several other key study design parameters, such as length of the recall period, method for estimating network size, and transit hubs (e.g., ports, taxi stands) for sampling respondents.

Our formative fieldwork was conducted in two stages. In the first stage, we conducted eight two-hour focus groups, each with four to eight participants. Our focus groups were split up by age and gender to maximize participant participation. For instance, if both younger and older men were placed in a group, cultural norms would dictate that only the older men would dominate discussions. We conducted four focus groups in the relatively urban Kalemie City and four focus groups in a rural village of Tabac Congo.

In these focus groups, we asked respondents a series of open-ended questions on how they learned about deaths in their community. Using a translator, we conducted the interviews in either French and Swahili depending on group preference. We used the scripted questions below, probing or asking follow-up questions as necessary:

1. How do people in your community learn about deaths? How do you personally learn about deaths?

2. Generally, how long after a death does it take to learn about it?

3. Do you learn about deaths from in-person conversations? From phone calls? Social media? Text message?

4. How well-informed are people about the details of the death? (Age/sex/cause/etc.)

5. Are deaths stigmatized at all? Is there any reason people would not report deaths?

Next, we asked a series of questions about different candidate personal networks. We tested different social ties, including (i) people you have had a meal with in the past year; (ii) people you talk to once a week; (iii) blood-related kin; (iv) immediate neighbors; and (v) acquaintances you talked to in the last year. For each tie definition, we asked questions on the following topics:

1. Under [tie definition], can you directly count how many people you know? If not, can you guess how many people you know under [tie definition]? How confident do you feel in your answer?

2. Under [tie definition], what kind of people are you connected to? Similar people? Random other people?

3. How much do you know about other people you are connected to through [tie definition]?

4. Would you know if someone in this network passed away in the past one month? Three months? Six months?

5. For people you are connected to by [tie definition], are you more likely to know whether certain groups of people died (men vs. women, young vs. old)?

Respondents were prompted to first directly answer the questions and then to engage in a broader discussion with other respondents. From these discussions, several insights emerged. First, respondents in nearly all focus groups reported learning about deaths predominantly from word of mouth. Social media, especially commemorative posts on WhatsApp status updates, was another common way for respondents to learn about deaths in urban, but not

rural, settings. In every focus group, respondents reported a high degree of certainty when reporting on deaths occurring in their extended kin or their immediate neighbors.

Respondents often gave nonsensically large answers when they were asked how many people they had a meal with in the past month or how many people they knew in groups of known size (e.g., how many teachers do you know?). This suggested that respondents were better able to count the number of people they were connected to in stronger social connections, such as blood-related kin.

The formative fieldwork also gave us the opportunity to gain insight into several other key study parameters, including the recall period. While in some humanitarian emergencies, circumstances may dictate the length of the recall window (e.g., the month directly following an earthquake), in more protracted humanitarian emergencies, this is a parameter researchers can vary. The choice of a recall period is important, as a recall period that stretches too far into the past may reduce respondents' ability to accurately recall and report about deaths. On the other hand, asking about too short a recall period may result in not enough information about deaths being collected to accurately estimate death rates.

S1.2 Insights from formative fieldwork

The qualitative data suggested that it would be valuable to use a significant and memorable reference event to start the recall period. Such locally recognizable events help respondents more accurately recall dates of death or approximate periods when deaths occurred; studies have used New Year's Day, Ramadan, and even the death of a prominent political figure [2]. Our qualitative research indicated that New Year's Day was a very salient event in this setting, helping respondents accurately determine whether a death occurred before or after this date. Based on this finding, we selected New Year's Day, January 1st, 2023, as our reference event.

These qualitative data also suggested that respondents were able to accurately report about deaths occurring in their extended kin network and their network of immediate neighbors. We used these insights to draft a preliminary set of survey questions and conducted 18 individual cognitive interviews. In these individual interviews, we asked respondents to talk out loud through answers and explain their rationale for their answers. This led to a

series of minor wording changes and clarifications of definitions (e.g., being more explicit in our wording that kin only includes blood relatives). For example, to help respondents count the number of people they were connected to in these networks, we broke down categories into smaller groups (e.g., number of female cousins age 0–4, number of male cousins age 0–4, number of female cousins age 5–18, number of male cousins age 5–18, number of female cousins 18+, number of male cousins 18+).

Respondents reported being able to accurately report on the size of their kin and immediate neighbor networks using this approach; this suggests that their total network size could be estimated using an approach called the *summation method*, which asks respondents to report on the number of people they are connected to in specific discrete categories and then sums those reports up to get an estimate of total personal network size [3, 4]. The advantage of the summation method is that it helps break down a personal network such as "extended kin" into subgroups that are easier to count. In the context of this study, it was particularly helpful as respondents often struggled to count the number of people they were connected to in larger groups.

To summarise, we conducted formative field work in our focal health zones. This formative research revealed that respondents were confident they could accurately report on deaths occurring after New Year's in two of their personal networks: their extended kin network and their immediate neighbor network. Further, respondents reported being able to confidently report on deaths after New Year's and the size of extended kin networks and immediate neighbor networks using the summation method.

S2 Study design

S2.1 Quota survey

The quota survey was designed to test our new network-based approach by asking respondents to report on mortality in their kin network and in their neighbor network. The frame population—the universe of people eligible to respond to the survey—was all adults over age 18 who reported living in one of the three focal health zones: Nyunzu, Nyemba, and

Kalemie. We used a non-probability, quota-based sampling strategy to sample respondents at major transit hubs, such as ports, markets, taxi stands, foot paths, and health clinics in Kalemie City, the capital of Tanganyika Province. We chose this diverse set of transit hubs in hopes of sampling as representative a sample as possible. The number of interviews per site type is shown in Table S1.

Site type	n
Health facility	358
Market	833
Other transport	1136
Port	113
Taxi	211

Table S1: Study Sites

Our quotas specified a target number of respondents in cells defined by gender and by all of the health areas[1] that lie in Nyunzu, Nyemba, and Kalemie. These quotas were established based on available population data from the Ministry of Health using vaccination campaign micro-planning information.

A total of 2,526 interviews were conducted from March 1st, 2023 to June 29th, 2023. We emphasize that we recommend using probability sampling wherever possible; however, in this study our goal was to explicitly test this non-probability quota sampling design, because it is the kind of data collection strategy that would be feasible during a humanitarian crisis.

The quota survey proceeded as follows (see Section S6 for the full survey instrument). After obtaining informed consent, respondents were asked a series of screener questions to determine eligibility for the survey. If respondents were eligible to participate in the survey based on quotas, they first answered a series of questions about their demographic and socioeconomic characteristics. Respondents were asked about age, sex, education level, occupation, and a set of questions to construct a wealth index: owning a bed, owning a radio, material of the exterior walls of their dwelling unit, and primary fuel used for cooking.

Next, respondents were asked to report on deaths in their kin and neighbor networks in

[1] There are currently 26 provinces in DRC. These provinces are subdivided into a total of 519 health zones (also called Zones de Santé), and each health zone is further divided into Health Areas (also called Aires de Santé). See https://data.humdata.org/dataset/drc-health-data for more information.

separate modules. We selected these two networks based on a series of focus groups and cognitive interviews conducted to determine the specific social ties that respondents could accurately report on. The order of the kin and neighbor modules were randomly assigned to allow us to assess potential question ordering effects.

Respondents reported on the number of connections they had in different subgroups (e.g., "How many male cousins do you have under the age of 5?"). We then immediately asked respondents to report on the number of deaths in these groups. In both modules, we broke these questions into finer subcategories to reduce cognitive load on respondents and improve the accuracy of reporting. After completing the network modules, respondents were asked a series of questions about births, migration, measles, and cholera in their personal networks. Finally, if respondents reported a death in their household, they were asked a series of detailed follow-up questions about the timing and an abbreviated WHO verbal autopsy.

S2.2 Recall period

Respondents were asked to report on deaths occurring between the reference date, January 1st, 2023, and the interview date. Since the network survey was in the field for four months, a respondent's recall period varied depending on their interview date. Our estimator compensates for different recall periods among respondents by including a term E_i, representing the total exposure days each respondent reported about their personal networks (Equation 3). We estimate the total amount of exposure reported by a respondent, in person-days, by taking the product of the length in days of the respondent's recall period and their personal network size.

Notably, this rolling recall period resulted in more reports about deaths and exposure at the beginning of the observation window and fewer towards the end. For instance, respondents interviewed in March could not report on deaths in June. We pooled information on all deaths and exposure from January 1st, 2023, to June 29th, 2023, assuming that the CDR remained constant throughout the period. This assumption seems reasonable, because we found negligible changes in estimated death rates over time. However, in different contexts with a stronger time trend in mortality, researchers might need to produce separate estimates for shorter time periods and average them together.

S2.3 Crude death rate units

We chose to express our CDR as deaths per 10,000 people per day. This contrasts with units more commonly used in demography: deaths per 1,000 people per year. To convert the CDRs reported in this paper to units of deaths per 1,000 people per year, simply multiply by 36.5.

1. As there are 365 days in a year, to convert from a daily rate to an annual rate, we multiply the CDR by 365.

2. The humanitarian CDR is expressed per 10,000 people, whereas the demographic CDR is expressed per 1,000 people. To account for this, we divide by 10.

The conversion factor is therefore calculated as:

$$\text{Conversion factor} = \frac{365 \text{ days per year}}{10} = 36.5 \tag{S1}$$

The rationale for expressing the CDR in these units is twofold. First, conditions in humanitarian disasters can fluctuate significantly on a daily basis, and so CDRs are often calculated for a time periods much shorter than a year (in contrast to conventional demographic CDRs, which are typically calculated for a year); estimating mortality over shorter time periods can capture these fluctuations better than an annual measure [5]. Second, this daily CDR is used as the basis for defining humanitarian emergencies; for example, the Center for Disease Control (CDC) defines a humanitarian crisis as more than 1 death per 10,000 persons per day [2].

S2.4 Mortality clustering within social networks

Mortality clustering—the non-random concentration of deaths within specific groups, such as households, social networks, and villages—has potential implications for the network survival method estimates presented in this paper. In certain settings, mortality may exhibit stronger clustering within extended networks than within households. For instance, for certain infectious diseases, deaths may cluster among immediate neighbors who spread the

disease to each other. Whether clustering is greater within extended networks or within households will ultimately be context-specific.

This potential clustering should not introduce bias into the point estimates but does lead to greater variability and increased uncertainty. Our bootstrap resampling procedure explicitly captures this uncertainty and accounts for the clustering of mortality within networks. We recommend that all future studies incorporating the network survival method implement a similar bootstrap procedure for uncertainty quantification.

S2.5 Probability survey

In addition to the quota survey, we collected a probabilistic, retrospective household mortality survey (probability survey) administered between July 24th, 2023 and September 2nd, 2023. We sample 2,785 households from our focal health zones of Nyunzu, Nyemba, and Kalemie. The sampling frame was constructed from population data from the Ministry of Health derived from vaccination campaign micro-planning information. Using these population data, we defined primary sampling units, generally at the village level. We randomly sampled 38 primary sampling units in Kalemie,[2] 40 primary sampling units in Nyunzu, and 44 primary sampling units in Nyemba. Within primary sampling units, households were selected using random sampling. Within households, the household head, or in their absence another adult over the age of 18, was surveyed. The household survey asked detailed information about deaths occurring within their household after January 1st, 2023 and the full set of network method questions. If respondents reported a death within their own household, a supervisor then followed up the same day with a verbal autopsy questionnaire to collect detailed information about the cause of the death.

We consider the estimate from the probability survey to be valuable as a comparator, but we note that it is not a gold standard. Like all estimates based on a retrospective household survey, our estimates may be prone to different sources of error, including sampling error, response errors, and frame errors that may affect the accuracy of the household-based estimate [6–9].

[2]Due to insecurity in parts of Kalemie, we were only able to access 38 PSUs in the Kalemie health zone because of security issues in the southern areas at the time of the survey.

S2.6 Weighting strategies

For our quota survey, we used a non-probability quota sample with quotas based on gender and health area. This design led our respondents to match the overall gender and geographic distribution of our target population very closely. However, because our design did not choose respondents probabilistically, there are still ways that selection bias may affect the composition of our survey respondents. Specifically, our sample overrepresents higher SES individuals and middle-aged respondents (Figure S1). Given the observable selection into our sample, we develop a few different weighting strategies to adjust for potential selection into our network survey sample.[3]

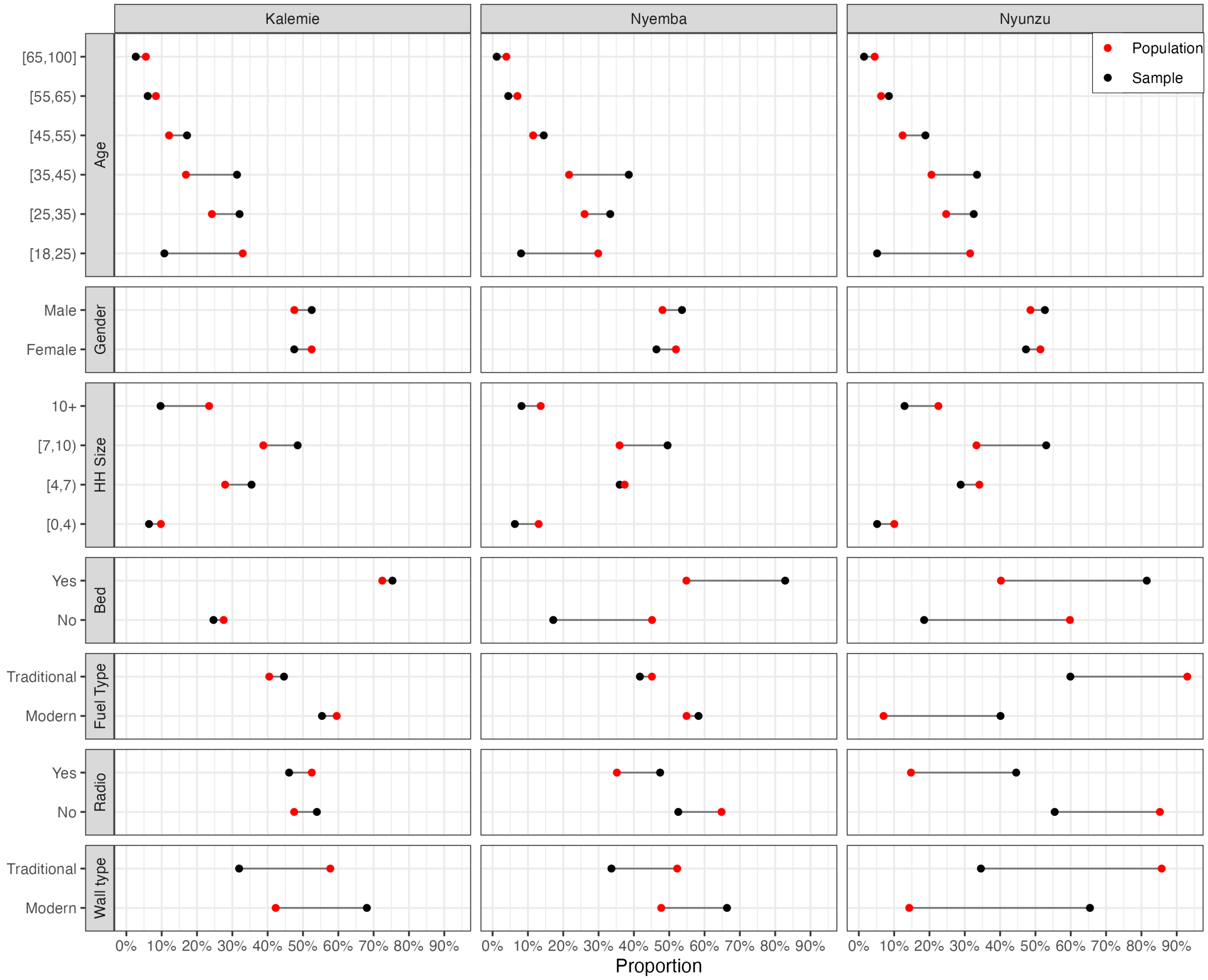

Figure S1: Difference in respondent composition between quota and household samples.

[3]The framework we adopt for inference from a non-probability sample is sometimes called *quasi-randomization* [10].

We construct three different sets of estimates using different weighting strategies imitating different data availability settings. Our first set of estimates are unweighted. This unweighted strategy relies exclusively on our quota sample based on geographic region (health area, the geographic unit beneath health zones) and gender. This gives us a baseline set of estimates not adjusting for any of the selection into the sample.

Our second set of estimates imitates a setting where no auxiliary data specific to our setting is available to help construct survey weights. Instead, we use modeled data from the WorldPop gridded population estimates—which are available all over the world—to construct poststratification weights [11]. We use the 2020 age and gender-structured, gridded cells with a resolution of 100m. We construct weighting targets by taking the intersection of these gridded cells with administrative boundaries for each of the three health zones using administrative boundaries from the GADM project. We then construct post-stratification weights on the following cells: age (18–24, 25–34, 35–44, 45–54, 55–64, 65+), gender (female and male), and health zone (Nyunzu, Nyemba, and Kalemie).

For our final set of estimates, we construct survey weights using logistic regression to model inclusion probability. We use our household survey, which in this setting represents the most accurate set of reference estimates. There was no other representative household survey large enough to serve as a reliable reference survey. In other settings, a recent household survey, such as a Demographic and Health Survey (DHS), might serve as a reliable reference. To take advantage of this, researchers must design their non-probability survey instrument carefully to ensure harmonization with the reference survey. Question wording for sociodemographic and household questions was identical between the quota and probability surveys.

Specifically, we combined together our quota and household surveys, and fit models to estimate inclusion probability:

$$w_i = \frac{1}{\hat{P}(S_i = 1)} \tag{S2}$$

where w_i is a weight defined as the inverse probability of being included in the sample ($S_i = 1$). We estimate three separate regression models, one for each health zone, using the following specification:

$$\text{logit}(\Pr(\text{inclusion} = 1|\mathbf{X})) = \beta_0 + \beta_{(\text{gender})} + \beta_{(\text{age class})} + \beta_{(\text{hh size})} \\ \beta_{(\text{radio})} + \beta_{(\text{bed})} + \beta_{(\text{wall material})} + \beta_{(\text{modern fuel type})} + \\ \beta_{(\text{hh count age 0–4})} + \beta_{(\text{hh count age 5–17})} + \beta_{(\text{hh count age 18+})} \tag{S3}$$

where inclusion denotes the dependent variable indicating whether an individual is included within a specific zone. Independent variables comprise both continuous and categorical predictors: gender (male, female), age class (18–24, 25–34, 35–44, 45–54, 55–64, 65–100), household size (0-3, 3-6, 7+), household possession of a radio, household possession of a bed, household having a modern constructed wall type, household's primary fuel source being modern, and number of household members under age 5, between age 5 and 18, and over age 18. The regression coefficients and goodness of fit statistics are reported in Table S2.

	Kalemie	Nyemba	Nyunzu
(Intercept)	0.044***	0.016***	0.004***
	(0.012)	(0.005)	(0.002)
Gender (Male)	1.166	1.140	0.894
	(0.108)	(0.106)	(0.116)
Age class [25,35)	3.077***	3.473***	5.341***
	(0.445)	(0.536)	(1.312)
Age Class [35,45)	5.882***	5.009***	7.884***
	(0.889)	(0.791)	(1.980)
Age Class[45,55)	5.112***	4.405***	12.363***
	(0.860)	(0.803)	(3.318)
Age Class[55,65)	2.943***	2.579***	16.544***
	(0.618)	(0.617)	(5.279)
Age Class[65,100]	2.018*	1.715	3.865**
	(0.558)	(0.652)	(1.798)
Household Size Size 4–7	0.879	1.101	0.796
	(0.197)	(0.256)	(0.273)
Household Size 7–10	0.718	1.076	0.810
	(0.181)	(0.283)	(0.309)
Household Size 10+	0.194***	0.444**	0.192***
	(0.056)	(0.135)	(0.080)
Owns radio	0.645***	1.356**	2.411***
	(0.067)	(0.136)	(0.354)
Owns Bed	1.258	3.790***	3.300***
	(0.150)	(0.440)	(0.483)
Modern House Material	4.189***	2.325***	6.918***
	(0.425)	(0.259)	(0.946)
Use Modern Fuel	0.821	0.484***	4.129***
	(0.090)	(0.056)	(0.681)
Under 5 Count (1)	1.674***	2.314***	2.095***
	(0.216)	(0.301)	(0.410)
Under 5 Count (2+)	4.987***	3.621***	8.318***
	(0.646)	(0.474)	(1.643)
Age 5–18 Count (1)	0.846	0.715	1.133
	(0.161)	(0.143)	(0.333)
Age 5–18 Count (2+)	0.900	1.227	1.787*
	(0.165)	(0.241)	(0.485)
Age 18+ Count (2+)	0.965	0.999	0.377***
	(0.217)	(0.249)	(0.110)
Num.Obs.	3250	3087	3210
AIC	2417.6	2232.4	1686.0
BIC	2533.2	2347.1	1801.5
RMSE	0.38	0.39	0.28

*p <0.05, ** p <0.01, *** p <0.001

Table S2: Logistic regression predicting odds of inclusion in the probability sample. Coefficients report odds ratios.

For the probability survey, we do not use survey weights. The probability sample was intended to produce a self-weighting sample, and in the absence of any other high-quality assessment, our probability survey is the most reliable source of population composition

estimates available. In settings where high-quality auxiliary data is available, we recommend reweighting the probability sample to account for non-response and other biases.

S2.7 Blended network estimates

We produced separate estimates using reports about neighbor and kin networks. In addition, we use a blended estimator to produce a combined estimate based on both the kin and the neighbor network reports [12]. The advantage of this blended approach is that we expect it to produce smaller mean squared error (MSE) than either the kin or neighbor estimate alone, because the blended estimate is based on more information. But this comes at the cost of additional assumptions; see [12] for a detailed discussion.

The blended estimate is based on averaging together the estimate from each network in a principled way. Suppose we have two estimators for N, $\widehat{N}^A$ and $\widehat{N}^B$. We define the blended estimate with pooling weight θ as:

$$\underbrace{\widehat{N}}_{\text{Blended Estimator}} = \underbrace{\theta \widehat{N}^A}_{\text{Weighted Estimator A}} + \underbrace{(1-\theta)\widehat{N}^B}_{\text{Weighted Estimator B}} \tag{S4}$$

where $\theta \in \mathbb{R}$.

Given estimates of the sampling variance for the two estimates, and assuming that both estimators $\widehat{N}^A$ and $\widehat{N}^B$ are unbiased, we can calculate the weight $\theta^\star$ that minimizes the expected mean squared error as:

$$\theta^\star = \frac{\sigma_B^2 - \sigma_{AB}}{\sigma_A^2 + \sigma_B^2 - 2\sigma_{AB}}, \tag{S5}$$

where σ_A^2 is the sampling variance of estimator $\widehat{N}^A$, σ_B^2 is the sampling variance of estimator $\widehat{N}^B$, and $\sigma_{AB} = cov(\widehat{N}^A, \widehat{N}^B)$ is the covariance of estimator $\widehat{N}^A$ and $\widehat{N}^B$. The blending weights given by Equation S5 are the ones we use to blend estimates in the main text; a full derivation is in Section S2.7.1.

Future studies may have more information about the bias of estimators, perhaps from validation studies. In that case, it would be helpful to have weights that can be used to blend biased estimates together, accounting for the bias. Section S2.7.2 derives another optimal

weight in this more general situation:

$$\theta^\star = \frac{\sigma_B^2 - \sigma_{AB} + \beta_B(\beta_B - \beta_A)}{\sigma_A^2 + \sigma_B^2 - 2\sigma_{AB} + (\beta_A - \beta_B)^2}, \tag{S6}$$

where $\beta_A = \mathbb{E}[\widehat{N}_A - N]$ is the bias of estimator $\widehat{N}_A$ and $\beta_B = \mathbb{E}[\widehat{N}_B - N]$ is the bias of estimator $\widehat{N}_B$.

S2.7.1 Derivation of optimal weight for blended estimator assuming each estimator is unbiased

Here we will derive the blending weight $\theta^\star$ that minimizes the mean squared error (MSE) of our blended estimate (Equation S5). We consider two estimators for N, denoted $\widehat{N}^A$ and $\widehat{N}^B$, which will be combined with a pooling weight θ, as in Equation S4. We assume $\widehat{N}^A$ and $\widehat{N}^B$ are unbiased for N, meaning that

$$\mathbb{E}[\widehat{N}^A - N] = 0 \text{ and } \mathbb{E}[\widehat{N}^B - N] = 0.$$

The blended estimator with blending weight θ has MSE

$$MSE(\hat{N}) = \mathbb{E}\left[\left(\theta\widehat{N}^A + (1-\theta)\widehat{N}^B - N\right)^2\right]. \tag{S7}$$

In general, for a random variable X, we have $\mathbb{E}[X^2] = \mathbb{E}[X]^2 + \mathrm{Var}[X]$. Applying this relationship to the MSE, we obtain

$$MSE(\hat{N}) = \left(\mathbb{E}\left[\theta\widehat{N}^A + (1-\theta)\widehat{N}^B - N\right]\right)^2 + \mathrm{Var}\left[\left(\theta\widehat{N}^A + (1-\theta)\widehat{N}^B - N\right)\right]. \tag{S8}$$

By assumption, our estimators $\widehat{N}^A$ and $\widehat{N}^B$ are unbiased for N, so $\mathbb{E}\left[\left(\theta\widehat{N}^A + (1-\theta)\widehat{N}^B - N\right)\right] = 0$. This leaves us with just the variance term

$$MSE(\hat{N}) = \mathrm{Var}\left[\left(\theta\widehat{N}^A + (1-\theta)\widehat{N}^B - N\right)\right] = \mathrm{Var}\left[\left(\theta\widehat{N}^A + (1-\theta)\widehat{N}^B\right)\right]. \tag{S9}$$

This can be further simplified using properties of variance. If we let $\text{Var}\left[\widehat{N}^A\right] = \sigma_A^2$, $\text{Var}\left[\widehat{N}^B\right] = \sigma_B^2$, and $\text{Cov}\left[\widehat{N}^A, \widehat{N}^B\right] = \sigma_{AB}$, this will simplify to:

$$MSE(\hat{N}) = \text{Var}\left[\theta\widehat{N}^A\right] + \text{Var}\left[(1-\theta)\widehat{N}^B\right] + 2\text{Cov}\left[\theta\widehat{N}^A, (1-\theta)\widehat{N}^B\right] \tag{S10}$$

$$= \theta^2\text{Var}\left[\widehat{N}^A\right] + (1-\theta)^2\text{Var}\left[\widehat{N}^B\right] + 2\theta(1-\theta)\text{Cov}\left[\widehat{N}^A, \widehat{N}^B\right] \tag{S11}$$

$$= \theta^2\sigma_A^2 + (1-\theta)^2\sigma_B^2 + 2\theta(1-\theta)\sigma_{AB}. \tag{S12}$$

To find the blending weight $\theta^\star$ that is optimal in the sense that it minimizes the $MSE(\widehat{N})$, we will take the derivative of Equation S12 with respect to θ, set it equal to 0, and solve for the optimum, $\theta^\star$.

$$\begin{aligned}
\frac{\partial MSE(\hat{N})}{\partial\theta} &= 2\theta\sigma_A^2 - 2(1-\theta)\sigma_B^2 + 2\sigma_{AB} - 4\theta\sigma_{AB} = 0 \\
&\Leftrightarrow 2\theta\sigma_A^2 - 2\sigma_B^2 + 2\theta\sigma_B^2 + 2\sigma_{AB} - 2\theta\sigma_{AB} = 0 \\
&\Leftrightarrow \theta\left(\sigma_A^2 + \sigma_B^2 - 2\sigma_{AB}\right) = \sigma_B^2 - \sigma_{AB} \\
&\Leftrightarrow \theta^\star = \frac{\sigma_B^2 - \sigma_{AB}}{\sigma_A^2 + \sigma_B^2 - 2\sigma_{AB}}
\end{aligned}$$

This derivation goes beyond past results on blended estimates from Feehan et al. [12] by relaxing an important assumption: that both estimates are independent.

S2.7.2 Derivation of optimal weight for blended estimator assuming each estimator is biased

In this section, we provide an expression for the blending weight that minimizes MSE if the estimators are biased. Although we do not apply these results in our study, they may prove useful in future studies where bias has been measured, perhaps using validation study designs. The results in this section nest the results in the previous section in the special case where the bias of the two estimators is 0.

Assume the same setup as in Section S2.7.1, except that $\widehat{N}^A$ and $\widehat{N}^B$ may be biased. Let

the bias of $\widehat{N}^A$ be $\beta_A = \mathbb{E}[\widehat{N}^A - N]$ and let the bias of $\widehat{N}^B$ be $\beta_B = \mathbb{E}[\widehat{N}^B - N]$.

Equation S8 showed that the MSE of the blended estimator with blending weight θ can be written

$$MSE(\hat{N}) = \left(\mathbb{E}\left[\theta\widehat{N}^A + (1-\theta)\widehat{N}^B - N\right]\right)^2 + \text{Var}\left[\left(\theta\widehat{N}^A + (1-\theta)\widehat{N}^B - N\right)\right]. \quad \text{(S13)}$$

However, unlike the derivation in the previous section, here we do not assume that the two estimators $\widehat{N}^A$ and $\widehat{N}^B$ are unbiased. This means that the first term of Equation S13 is not zero. Instead, it is the squared bias of the blended estimator:

$$\begin{aligned}
\left(\mathbb{E}\left[\theta\widehat{N}^A + (1-\theta)\widehat{N}^B - N\right]\right)^2 &= \left(\mathbb{E}\left[\theta\widehat{N}^A - \theta N + (1-\theta)\widehat{N}^B - (1-\theta)N\right]\right)^2 && \text{(S14)}\\
&= \left(\theta\mathbb{E}\left[\widehat{N}^A - N\right] + (1-\theta)\mathbb{E}\left[\widehat{N}^B - N\right]\right)^2 && \text{(S15)}\\
&= \left(\theta\beta_A + (1-\theta)\beta_B\right)^2. && \text{(S16)}
\end{aligned}$$

The second part of Equation S13 is the variance of the blended estimator; this term is unchanged. As Section S2.7.1 showed, the variance term can be written

$$\text{Var}\left[\left(\theta\widehat{N}^A + (1-\theta)\widehat{N}^B - N\right)\right] = \theta^2\sigma_A^2 + (1-\theta)^2\sigma_B^2 + 2\theta(1-\theta)\sigma_{AB}. \quad \text{(S17)}$$

Combining the squared bias and the variance using Equation S13, we find that the MSE will be

$$\begin{aligned}
MSE &= \left(\mathbb{E}\left[\theta\widehat{N}^A + (1-\theta)\widehat{N}^B - N\right]\right)^2 + \text{Var}\left[\left(\theta\widehat{N}^A + (1-\theta)\widehat{N}^B - N\right)\right] && \text{(S18)}\\
&= \theta^2\sigma_A^2 + (1-\theta)^2\sigma_B^2 + 2\theta(1-\theta)\sigma_{AB} + \left(\theta\beta_A + (1-\theta)\beta_B\right)^2. && \text{(S19)}
\end{aligned}$$

To find the value of θ that minimizes the MSE, we take the derivative with respect to θ, set it equal to zero, and solve for $\theta^\star$:

$$\frac{\partial MSE(\hat{N})}{\partial \theta} = 2\theta\sigma_A^2 - 2(1-\theta)\sigma_B^2 + 2\sigma_{AB} - 4\theta\sigma_{AB} + 2(\theta\beta_A + (1-\theta)\beta_B)(\beta_A - \beta_B)0 \quad \text{(S20)}$$

$$= 2\theta\sigma_A^2 - 2\sigma_B^2 + 2\theta\sigma_B^2 + 2\sigma_{AB} - 4\theta\sigma_{AB} + 2\theta\beta_A^2 + 2\beta_A\beta_B - 2\theta\beta_A\beta_B - 2\theta\beta_A\beta_B - 2\beta_B^2 + 2\theta\beta_B^2 \quad \text{(S21)}$$

$$= 2\theta\left(\sigma_A^2 + \sigma_B^2 - 2\sigma_{AB} + \beta_A^2 - 2\beta_A\beta_B + \beta_B^2\right) - 2\left(\sigma_B^2 - \sigma_{AB} - \beta_A\beta_B + \beta_B^2\right). \quad \text{(S22)}$$

Setting this expression equal to 0 and solving for the minimizer, $\theta^\star$, we have

$$\theta^\star\left(\sigma_A^2 + \sigma_B^2 - 2\sigma_{AB} + \beta_A^2 - 2\beta_A\beta_B + \beta_B^2\right) = \sigma_B^2 - \sigma_{AB} - \beta_A\beta_B + \beta_B^2$$

$$\iff \theta^\star = \frac{\sigma_B^2 - \sigma_{AB} + \beta_B(\beta_B - \beta_A)}{\sigma_A^2 + \sigma_B^2 - 2\sigma_{AB} + \beta_A^2 - 2\beta_A\beta_B + \beta_B^2}$$

$$\iff \theta^\star = \frac{\sigma_B^2 - \sigma_{AB} + \beta_B(\beta_B - \beta_A)}{\sigma_A^2 + \sigma_B^2 - 2\sigma_{AB} + (\beta_A - \beta_B)^2}.$$

Note that, plugging in $\beta_A = 0$ and $\beta_B = 0$, we recover the weight derived for unbiased estimators in the previous section.

We can confirm that $\theta^\star$ is a minimum by differentiating Equation S22 again to obtain

$$\frac{\partial^2 MSE(\hat{N})}{\partial \theta^2} = 2\left(\sigma_A^2 + \sigma_B^2 - 2\sigma_{AB} + \beta_A^2 - 2\beta_A\beta_B + \beta_B^2\right) \quad \text{(S23)}$$

$$= 2(\beta_A - \beta_B)^2 + 2(\sigma_A^2 + \sigma_B^2 - 2\sigma_{AB}). \quad \text{(S24)}$$

When Equation S24 is greater than 0, $\theta^\star$ will be a minimum. The first term in parentheses, $(\beta_A - \beta_B)^2$ will be greater than zero except in the special case where the bias of the two estimators is identical, i.e., $\beta_A = \beta_B$, which will make the term zero. The second term in parentheses, $\sigma_A^2 + \sigma_B^2 - 2\sigma_{AB}$ is equal to the variance of the difference between the two estimators, $\text{var}(\widehat{N}_A - \widehat{N}_B)$. As a variance, this is always greater than or equal to zero, and

will equal zero only when $\widehat{N}_A = \widehat{N}_B$. Thus, we conclude that Equation S24 is greater than zero except for the pathological case where $\beta_A = \beta_B$ and $\widehat{N}_A = \widehat{N}_B$.

In our study, the $\theta^\star$ used for blending is always in $[0, 1]$, meaning that it will produce an estimated value in-between $\widehat{N}^A$ and $\widehat{N}^B$. We expect this to be true in most applied settings, but this is not guaranteed; future work could explore when the blending weight will be outside that range.

S2.8 Comparisons with other studies

The Jarrett et al. [9] study took place in the Fizi health zone in South Kivu, which is directly above our focal health zones (Figure S2). The study combined data from both a surveillance program and a retrospective household mortality study. The surveillance program had a recall period of November 1st 2011 to September 30th 2012; the presidential election took place on November 2011, making November a salient reference date. For brevity, we only discuss the mortality estimation component of the study.

The retrospective household survey took place from August 29th, 2012 to September 14th, 2012. The recall period was from November 1st, 2011 until the day of the interview, a period approximately equivalent to the surveillance program recall period. Any discrepancies between the household survey and the surveillance site (i.e., death event reported in one system and not in the other) was investigated in a re-evaluation process. In this re-evaluation, enumerators visited households and asked a series of questions to validate whether a reported event had actually occurred.

The study derived a gold-standard estimate, which used deaths that either (1) matched in both the household survey and surveillance systems or (2) were confirmed as a true death in the re-evaluation stage. The study found 23 true deaths and 38 false positive death reports in the household survey. Of these false positive reports, 12 deaths were outside of recall bounds, 18 deaths were not within the household, and 8 deaths were simply fabricated.

The magnitude of discrepancies is relevant to our study. The respondents sampled here, much like in our focal health zones, may have an incentive to report their situation as being particularly aid-worthy. While the study was conducted approximately 12 years before our study, such overreporting dynamics are also possible in our survey.

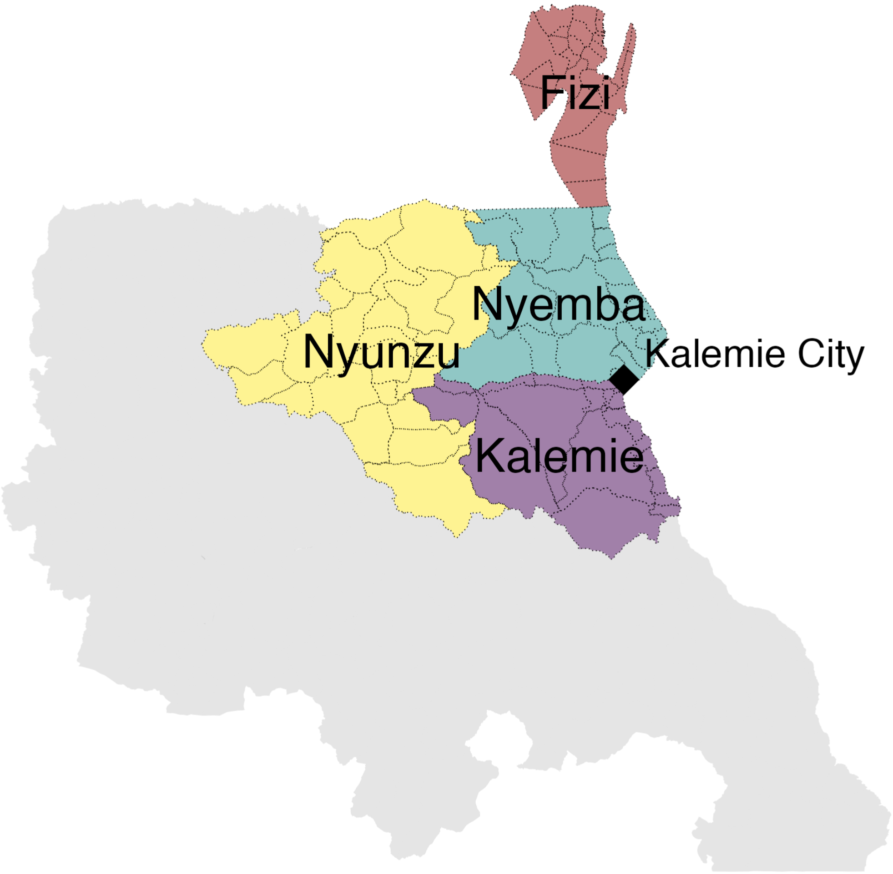

Figure S2: This map shows the geographic proximity of our focal health zones to Fizi, the territory considered in Jarrett et al.[9].

Another comparison is the household SMART Survey administered in November 2022 in the Kalemie Health Zone [13]. To facilitate a more direct comparison, we compare our estimates for the Kalemie Health Zone to the SMART Survey in Figure S3.

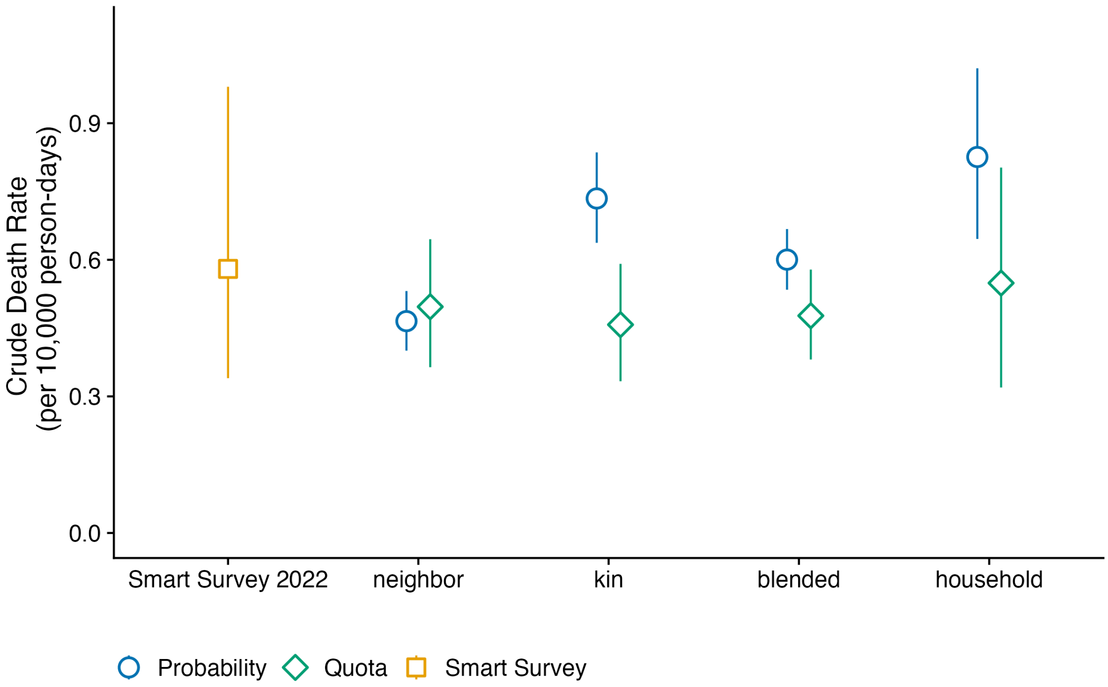

Figure S3: Direct comparison of estimates from our study to external estimates from a 2022 household SMART Survey.

S2.9 Investigating sources of discrepancies

To better understand the potential reasons for the discrepancy between the household and network estimates in our probability sample, we investigate two potential sources of bias: transmission error and strategic overreporting. We compare within the probability sample to control for differences due to sampling design (network-based estimates from probability and non-probability samples are very similar).

Transmission error refers to violations of the perfect visibility assumption—that is, respondents not knowing about a death in the network they are reporting on. In the context of this study, one candidate explanation is differential transmission error: respondents might have more accurate recall for household deaths compared to deaths in their broader social network. Specifically, there would need to be under-reporting of deaths in the neighbor and kin network due to respondents not knowing about deaths that had occurred—but not in their own household, leading to an underestimation of CDRs in the network estimates.

To get a better sense of the extent of transmission error, we compare the household

estimate (0.81) to the neighbor estimate (0.40). If we assume the household estimate is correct, respondents would need to miss reporting 51% of the deaths that occurred in their neighbor network. This seems implausible, given that our qualitative research indicates respondents were both (1) confident in their ability to know about and report deaths in neighboring households and (2) expressed no reluctance to report on their neighbors.

Another candidate explanation for this discrepancy is strategic overreporting of deaths in the household. It is possible that respondents in the probability survey are over-reporting deaths in attempts of making their situation appear more aid-worthy. If the kin CDR estimate is correct, how much strategic overreporting (i.e., false positives) of household deaths would be needed to get our household CDR estimate? For every real death, respondents would need to falsely report 1.05 additional deaths, meaning that only 48% of reported deaths actually occurred in the household during the reporting window. While high, this is substantially lower than the 72% false positive rate found in Jarrett et al. 2020 [9]. This suggests that strategic overreporting is plausible in this setting.

These two calculations give a rough sense of how plausible transmission error and strategic overreporting are as explanations for differences between the household and network estimates. We focused on extreme cases in which one factor alone affects one of the estimates at a time. But in reality, a complex combination of factors, including transmission error and strategic overreporting, could lead to errors in either survey-based estimate. Future work should focus on validation designs that compare mortality estimation methods in a setting where gold-standard death rates are available to better understand the properties of both estimators.

S2.10 Ethical considerations of collecting network survival data

In designing our survey, we recognized the ethical challenges associated with reporting deaths of individuals outside the household, given that household surveys typically assume a household member can ethically report on all members within the same household. To address these concerns, we implemented several safeguards to protect respondent ethical compliance:

1. All collected data were anonymized, and no personally identifiable information (e.g.,

names, street addresses) was gathered. Data were securely stored on password-protected laptops and released to the study team only after ensuring anonymity, eliminating any risk of re-identification.

2. Respondents were explicitly informed during the consent process that the survey would be asked about the deaths of neighbors and extended kin, ensuring their awareness and voluntary participation.

3. Our study received ethics approval from the UC Berkeley Institutional Review Board (IRB) and local IRB approval from the University of Kinshasa.

We encourage future researchers and practitioners to adopt similar ethical safeguards when applying the methods introduced in this study.

S3 Validity checks and internal consistency checks

S3.1 Network survival method: internal consistency checks

One advantage of the network method is its potential for partial self-validation. Certain relationships are naturally reciprocal, and we can use this expectation to check for consistency. For example, sibling relationships should be reciprocal. Assuming a perfect probability sample and accurate reporting, we would expect in aggregate, men in our sample would report the same number of connections to sisters as women would report connections to brothers.

As an internal validity check, we compare three relationships in Figure S4 we would expect to be reciprocal: parent child–relationships, sibling relationships by gender, and cousin relationships by gender. We restrict to reported adults over 18, as we only sample adults over aged 18. In our unweighted results, there are small differences: the total number of female reports to brothers are slightly greater than the total number of male reports to sisters. However, our inverse-probability weighted results show nearly perfect reciprocity across all three relationship comparisons. The close alignment between expected reciprocal relationships provides strong evidence for the internal validity and reliability of our network data.

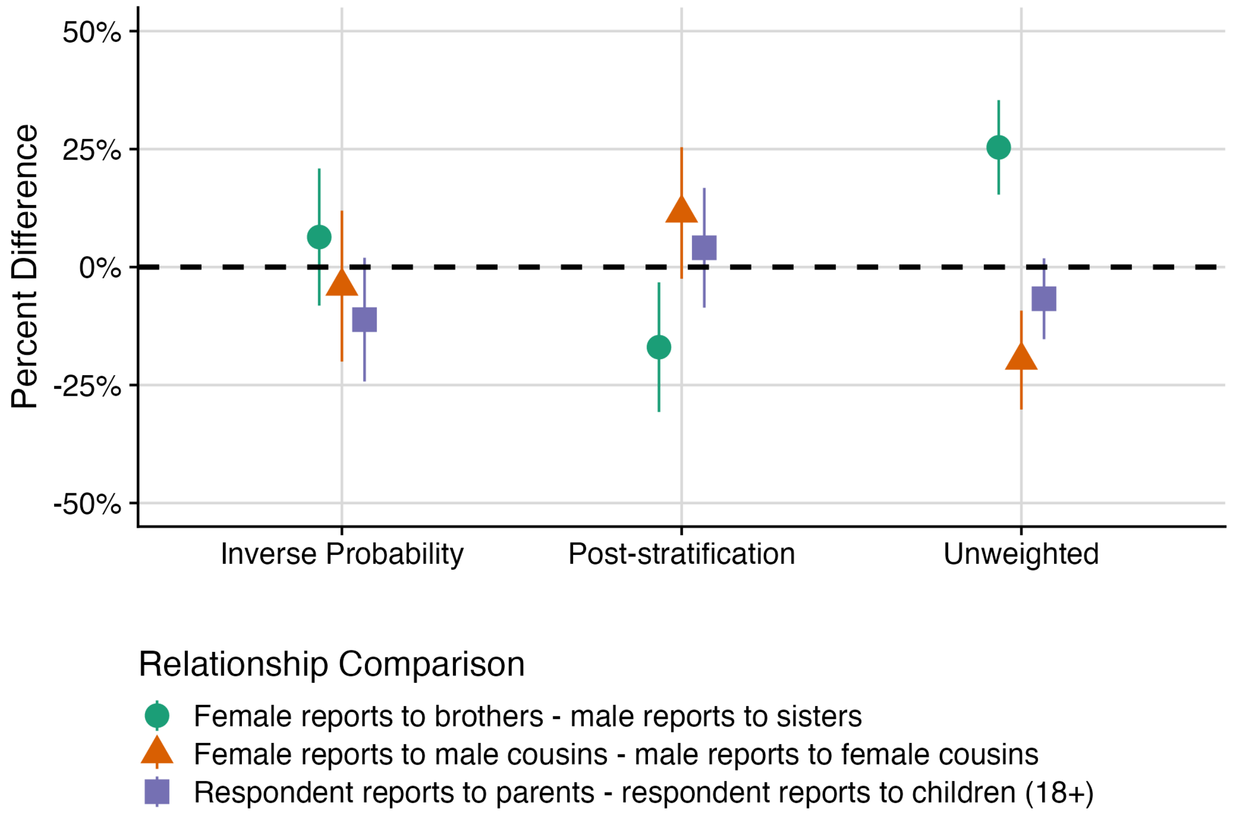

Figure S4: Internal validity checks.

S3.2 Robustness check: age compositions of networks

As a sensitivity check, we investigated the aggregate age composition of each network quota survey respondents report on. As shown in Figure S5, we benchmarked against age composition estimates obtained from the probability-based household rosters, which in this setting are the most reliable estimates available of household composition. We are restricted to the broad age categories of under age 5, 5–17, and 18 and over as we do not collect the exact age for each person the respondent reports on.

Our analysis reveals that, after applying survey weights, the age composition estimates from our quota sample closely match the benchmark data from the household rosters across both household and neighbor networks. This consistency check is reassuring, and suggests that respondents are accurately reporting the age composition of their neighbors. For the kin reports, we see that compared to household or neighbor reports, individuals aged 18 are

slightly overrepresented and 5–17 year olds are slightly underrepresented.

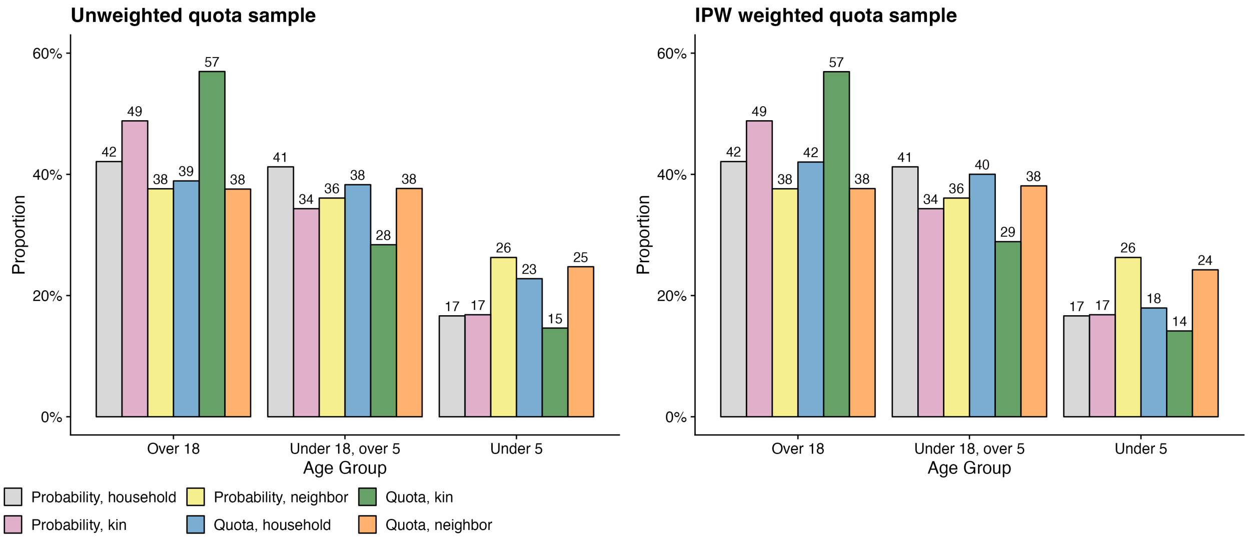

Figure S5: Age composition of different network reports in both quota and probability surveys.

S3.3 Validation check: module randomization

We randomized the order of the kin and neighbor network survival across surveys. As a validation check, we tested whether respondents reported differential network sizes or number of deaths depending on whether a module was administered first or second. Specifically, we wanted to confirm that respondents did not become fatigued taking the survey and report fewer deaths and/or smaller network sizes later in the interview. As shown in Figure S6, we find no statistically significant difference across survey modules for both the quota and the probability survey.

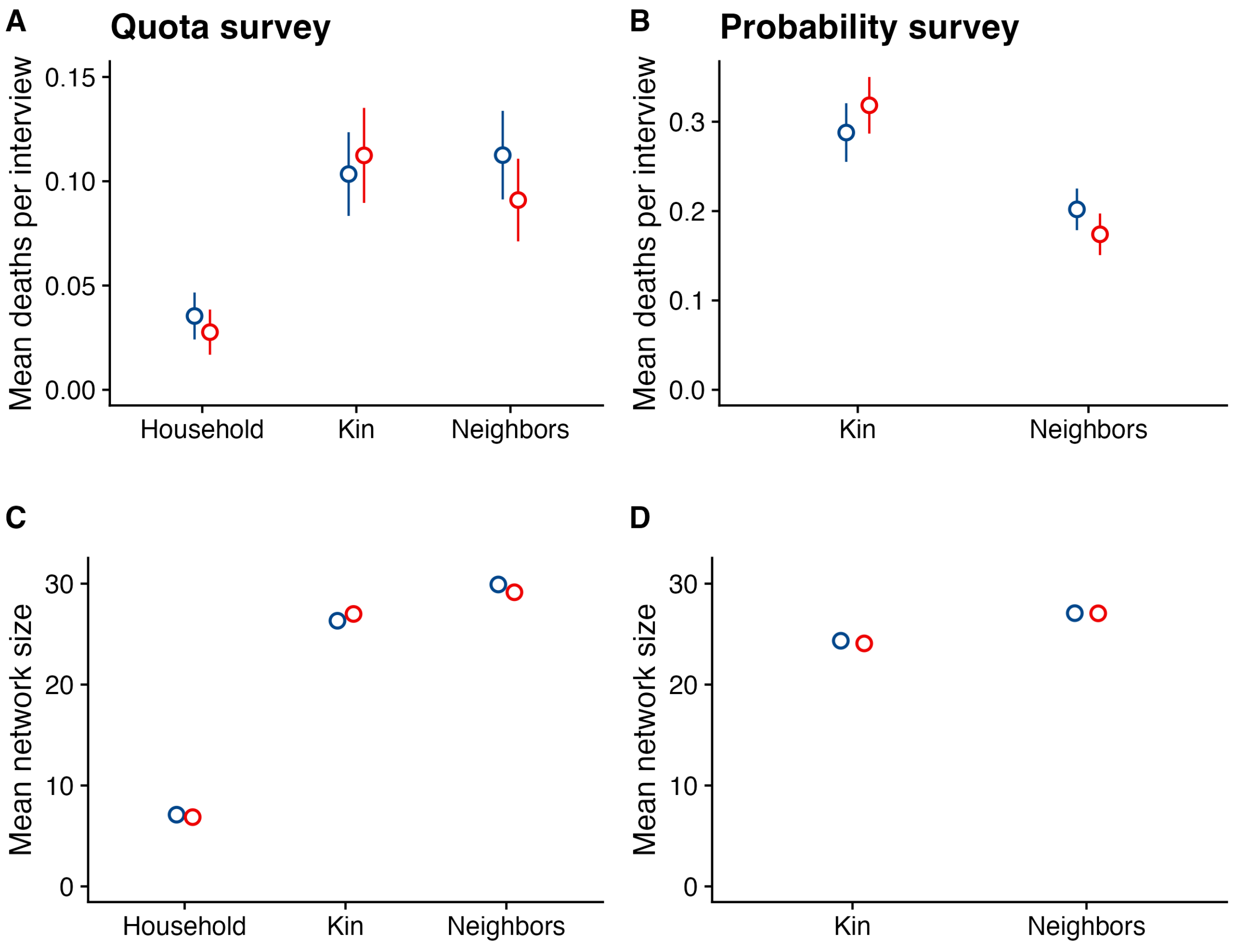

Figure S6: (*A*) and (*B*) show the mean deaths reported per interview by tie in the quota sample and probability sample depending on whether a module was randomly administered first (blue) or second (red). (*C*) and (*D*) similarly show the average network size by tie depending on randomized module order. We find no statistically significant or otherwise meaningful difference across survey modules for both the quota and the probability survey. **Notes:** In the probability survey, respondents reported on household deaths as part of a separate household module. This was not randomized, and always came before the network module. The probability survey has a larger number of mean reported deaths per interview than the quota survey as it asked about a substantially longer time window.

S4 Data processing

Additional Site in Nyunzu. To facilitate targeting the most remote areas in Nyunzu, we see set up a secondary sampling site in Nyunzu Town. Similar to Kalemie City, Nyunzu Town is an important town that attracts people from nearby villages. Some people from especially remote regions Health Areas of the Nyunzu Health Zone are quite far away from Kalemie City, making this a practical choice to help collect surveys.

This enumerator had little-to-no direct supervision from a field officer, and we still conducted the majority of our Nyunzu interviews (60%+) in Kalemie City. In our main analysis, we present estimates only based on interviews conducted in Kalemie City. As a robustness check, we regenerate weights and calculate our set of network estimates including the Nyunzu enumerator. As shown in Figure S7, there is no statistically significant difference between any estimates including and excluding the Nyunzu enumerator.

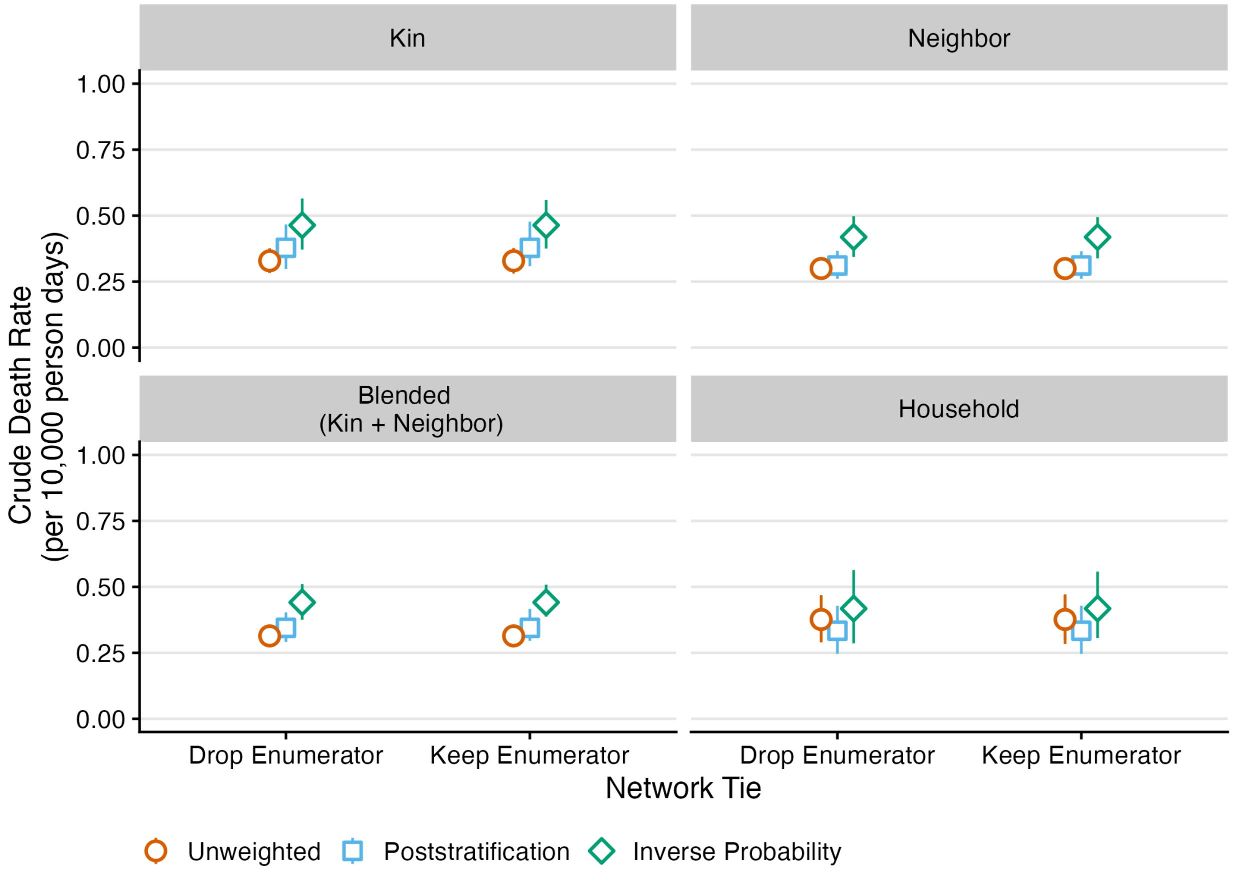

Figure S7: Difference in CDR estimates if Nyunzu Town enumerator is or is not included

S4.1 Missing data

Network method: numerator We drop any respondents with missing values on reported number of deaths. For all deaths, respondents were asked to give an exact date. If the respondents could not provide an exact day of death, they were asked to provide their best guess of the month in which the death occurred. We drop deaths that occurred before the beginning of our observation period, January 1st, 2023 ($N = 5$).

Network method: denominator We drop respondents who report missingness on questions about the size of their personal networks ($N = 18$). All respondents were asked to report on the closest five neighboring households by walking distance. In rare cases, respondents could not report accurately on the exact number of household members in all households, especially the fourth and fifth household. When respondents expressed uncertainty about the exact number of household members or gave a range of people living in the household, enumerators instructed the respondent to only report on their closest three households. These respondents were not dropped from the survey.

Weighting variables We drop records with missing values ($N = 3$) for sociodemographic and weighting variables, including owning a bed, type of cooking fuel, and livelihood. After dropping respondents with missing reported deaths, denominators, and socioedemographic characteristics, we were left with an analytic sample of 2,526 respondents.

S5 Additional results

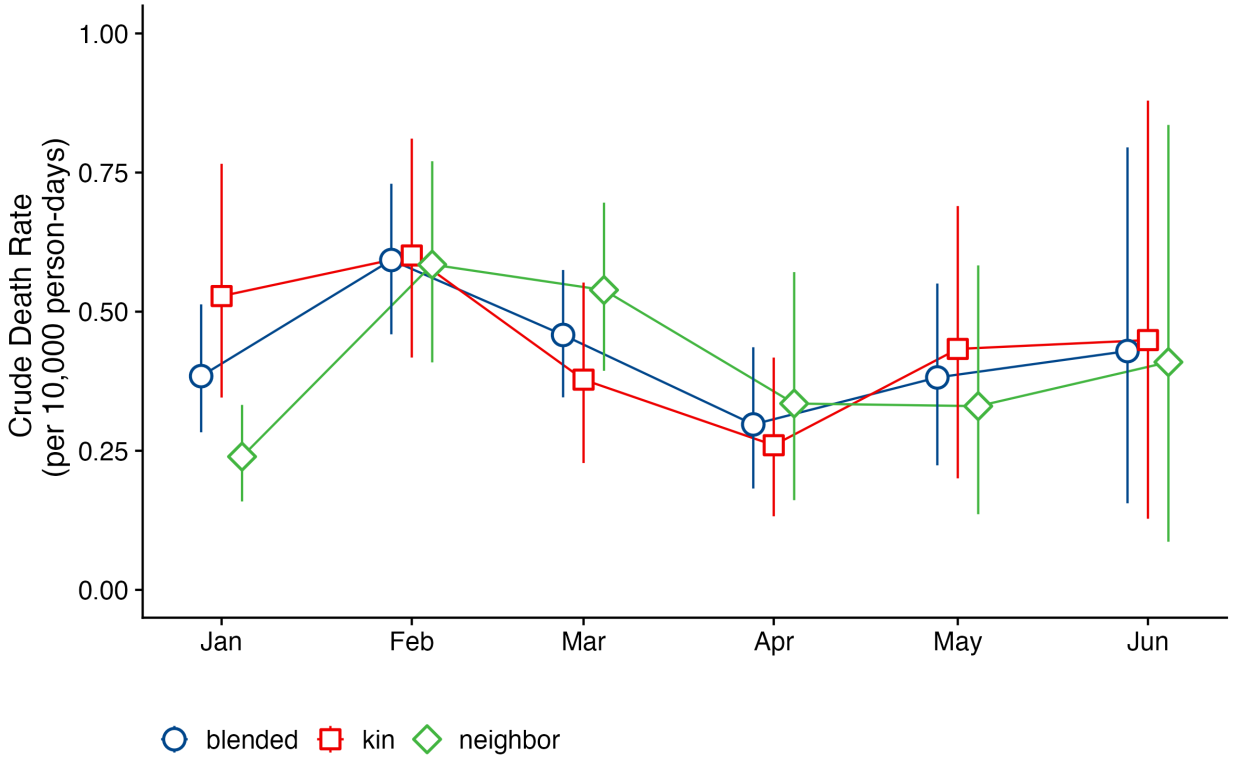

Figure S8: Quota sample estimates over time. Estimates are presented using inverse-probability weights.

Table S3: CDR estimates

Survey	Tie	Health Zone	Month	Weights	Death Rates	Lower	Upper
Probability	Kin	-	-	Unweighted	0.55	0.51	0.60
Probability	Neighbor	-	-	Unweighted	0.40	0.36	0.43
Probability	Blended	-	-	Unweighted	0.48	0.44	0.51
Probability	Household	-	-	Unweighted	0.81	0.71	0.92
Probability	Kin	Kalemie	-	Unweighted	0.73	0.64	0.84
Probability	Kin	Nyemba	-	Unweighted	0.50	0.42	0.58
Probability	Kin	Nyunzu	-	Unweighted	0.46	0.39	0.53
Probability	Neighbor	Kalemie	-	Unweighted	0.47	0.40	0.53
Probability	Neighbor	Nyemba	-	Unweighted	0.34	0.29	0.40
Probability	Neighbor	Nyunzu	-	Unweighted	0.39	0.33	0.44
Probability	Blended	Kalemie	-	Unweighted	0.60	0.53	0.67
Probability	Blended	Nyemba	-	Unweighted	0.42	0.37	0.48
Probability	Blended	Nyunzu	-	Unweighted	0.42	0.37	0.48
Probability	Household	Kalemie	-	Unweighted	0.83	0.65	1.02
Probability	Household	Nyemba	-	Unweighted	0.91	0.72	1.12
Probability	Household	Nyunzu	-	Unweighted	0.72	0.57	0.88
Quota	Kin	-	-	Unweighted	0.33	0.28	0.38
Quota	Neighbor	-	-	Unweighted	0.30	0.26	0.34
Quota	Blended	-	-	Unweighted	0.31	0.28	0.35
Quota	Household	-	-	Unweighted	0.38	0.29	0.47
Quota	Kin	-	-	Poststrat	0.38	0.30	0.47
Quota	Neighbor	-	-	Poststrat	0.31	0.26	0.37
Quota	Blended	-	-	Poststrat	0.34	0.29	0.40
Quota	Household	-	-	Poststrat	0.33	0.25	0.43
Quota	Kin	-	-	IPW	0.46	0.37	0.56
Quota	Neighbor	-	-	IPW	0.42	0.34	0.50
Quota	Blended	-	-	IPW	0.44	0.38	0.51
Quota	Household	-	-	IPW	0.42	0.29	0.56
Quota	Kin	Kalemie	-	IPW	0.46	0.33	0.59
Quota	Kin	Kalemie	-	Unweighted	0.37	0.29	0.46
Quota	Kin	Nyemba	-	IPW	0.30	0.22	0.40
Quota	Kin	Nyemba	-	Unweighted	0.30	0.24	0.38
Quota	Kin	Nyunzu	-	IPW	0.73	0.45	1.09
Quota	Kin	Nyunzu	-	Unweighted	0.30	0.22	0.40
Quota	Neighbor	Kalemie	-	IPW	0.50	0.36	0.65
Quota	Neighbor	Kalemie	-	Unweighted	0.33	0.27	0.41
Quota	Neighbor	Nyemba	-	IPW	0.27	0.20	0.35
Quota	Neighbor	Nyemba	-	Unweighted	0.29	0.23	0.35
Quota	Neighbor	Nyunzu	-	IPW	0.55	0.35	0.76
Quota	Neighbor	Nyunzu	-	Unweighted	0.27	0.20	0.33
Quota	Blended	Kalemie	-	IPW	0.48	0.38	0.58
Quota	Blended	Kalemie	-	Unweighted	0.35	0.30	0.41
Quota	Blended	Nyemba	-	IPW	0.28	0.22	0.36
Quota	Blended	Nyemba	-	Unweighted	0.30	0.25	0.35
Quota	Blended	Nyunzu	-	IPW	0.64	0.45	0.87
Quota	Blended	Nyunzu	-	Unweighted	0.29	0.23	0.35
Quota	Household	Kalemie	-	IPW	0.55	0.32	0.80
Quota	Household	Kalemie	-	Unweighted	0.50	0.34	0.68
Quota	Household	Nyemba	-	IPW	0.39	0.21	0.62
Quota	Household	Nyemba	-	Unweighted	0.42	0.27	0.58

Table S3: Death rate estimates for different health zones and months. *(continued)*

survey	social tie	health_zone	month	weights	death_rate	death_rate_lower	death_rate_upper
Quota	Household	Nyunzu	-	IPW	0.23	0.02	0.57
Quota	Household	Nyunzu	-	Unweighted	0.13	0.04	0.24
Quota	Kin	-	2023-01-01	IPW	0.53	0.35	0.77
Quota	Kin	-	2023-01-01	Unweighted	0.36	0.27	0.45
Quota	Kin	-	2023-02-01	IPW	0.60	0.42	0.81
Quota	Kin	-	2023-02-01	Unweighted	0.42	0.32	0.52
Quota	Kin	-	2023-03-01	IPW	0.38	0.23	0.55
Quota	Kin	-	2023-03-01	Unweighted	0.27	0.20	0.35
Quota	Kin	-	2023-04-01	IPW	0.26	0.13	0.42
Quota	Kin	-	2023-04-01	Unweighted	0.27	0.16	0.38
Quota	Kin	-	2023-05-01	IPW	0.43	0.20	0.69
Quota	Kin	-	2023-05-01	Unweighted	0.27	0.15	0.40
Quota	Kin	-	2023-06-01	IPW	0.45	0.13	0.88
Quota	Kin	-	2023-06-01	Unweighted	0.38	0.14	0.66
Quota	Neighbor	-	2023-01-01	IPW	0.24	0.16	0.33
Quota	Neighbor	-	2023-01-01	Unweighted	0.22	0.17	0.28
Quota	Neighbor	-	2023-02-01	IPW	0.58	0.41	0.77
Quota	Neighbor	-	2023-02-01	Unweighted	0.40	0.31	0.49
Quota	Neighbor	-	2023-03-01	IPW	0.54	0.39	0.70
Quota	Neighbor	-	2023-03-01	Unweighted	0.37	0.29	0.46
Quota	Neighbor	-	2023-04-01	IPW	0.33	0.16	0.57
Quota	Neighbor	-	2023-04-01	Unweighted	0.23	0.16	0.32
Quota	Neighbor	-	2023-05-01	IPW	0.33	0.14	0.58
Quota	Neighbor	-	2023-05-01	Unweighted	0.22	0.13	0.33
Quota	Neighbor	-	2023-06-01	IPW	0.41	0.09	0.84
Quota	Neighbor	-	2023-06-01	Unweighted	0.26	0.10	0.46
Quota	Blended	-	2023-01-01	IPW	0.38	0.28	0.51
Quota	Blended	-	2023-01-01	Unweighted	0.29	0.24	0.35
Quota	Blended	-	2023-02-01	IPW	0.59	0.46	0.73
Quota	Blended	-	2023-02-01	Unweighted	0.41	0.34	0.48
Quota	Blended	-	2023-03-01	IPW	0.46	0.35	0.57
Quota	Blended	-	2023-03-01	Unweighted	0.32	0.27	0.38
Quota	Blended	-	2023-04-01	IPW	0.30	0.18	0.44
Quota	Blended	-	2023-04-01	Unweighted	0.25	0.18	0.32
Quota	Blended	-	2023-05-01	IPW	0.38	0.22	0.55
Quota	Blended	-	2023-05-01	Unweighted	0.24	0.17	0.33
Quota	Blended	-	2023-06-01	IPW	0.43	0.16	0.80
Quota	Blended	-	2023-06-01	Unweighted	0.32	0.16	0.51

S6 Survey Instrument

The full survey instrument for the quota survey is shown below.

Network Method Survey Instrument

Section: Screening Script
I am _____, working for IMPACT Initiatives, a sister organization to ACTED, an international nonprofit organization working in this area. Together with the University of Kinshasa School of Public Health and University of California Berkeley, we are doing research on methods to improve reporting of deaths in the community to better inform the health department on the number and causes of death in this area. This information helps health actors plan and run health services for the population. We are approaching you today because you are coming from, or have information on, hard-to-reach communities in Tanganyika Province. Would you have 10-15 minutes today to answer some questions about births, deaths and other health events that have occurred in your community?<br><br>If yes, I would like to make sure that you are eligible before I give you more information about our work and invite you to take part in this study. May I ask, which Zone and Aire de Santé are you coming from today?<br><br>• [Visually assess the sex of the respondent]<br>• [Check against list if coming from a target area]<br><br>**Is the respondent eligible for the study?** [YES / NO]<br><br>[If not eligible for interview] Thank you for your time, however we do not need information from you today.<br>[END INTERVIEW]

Section: Informed Consent
[If they are eligible for interview] You are coming from an area where need information on the health situation of the population. Would you have 10-15 minutes to answer some questions for us about births, deaths or other health events that have occurred in your community?<br><br>If yes, I would like to give you some information about our work and invite you to take part in this study. If there is any part that you don't understand you can ask me to stop and I will take time to explain, or you can ask later. [APPLY INFORMED CONSENT FORM FOR NETWORK METHOD SURVEY]<br><br>** Has the respondent consented to participate? ** [YES / NO]<br><br>[If yes to consent] [Continue to section 1 below].<br>[If no to consent] Thank you for your time. [END INTERVIEW]

Section 1: Respondent Characteristics		
**S/No**	**Question**	**Choices**
Q1.1	What Zone de Sante are you coming from today?	[Select one – contextual list]
Q1.2	What Aire de Sante are you coming from today?	[Select one – contextual list]

Q1.3	What Village are you coming from today?	[Select one – contextual list]
Q1.4	Is [village_name] your place of usual residence?	1 = Yes<br>2 = No<br>8 = Don't know<br>9 = Prefer not to answer
Q1.5	What is the sex of the respondent?	1 = Male<br>2 = Female
Q1.6	What is the age of the respondent (in completed years)	Integer (completed years)
Q1.7	What is the marital status of the respondent?	1 = Single<br>2 = Married<br>3 = Divorced<br>4 = Widowed<br>5 = Other, please describe: _______________
Q1.8	What is the residency status of the respondent?	1 = Resident<br>2 = Internally Displaced Person (IDP)<br>3 = IDP Returnee<br>4 = Refugee Returnee<br>5 = Refugee
Q1.9	What is the highest level of education of the respondent?	1 = Pre-primary school<br>2 = Primary school<br>3 = Lower Secondary School<br>4 = Secondary School<br>5 = Post-secondary school<br>6 = Trade or professional school<br>7 = Religious school<br>8 = Don't know<br>9 = Prefer not to answer
Q1.10	What does the respondent do to make money or earn food for the household?	[select multiple – contextual list of livelihood activities]
Q1.11	What is the reason for the person's movement through town today?	1 = Transit to another location<br>2 = Access market<br>3 = Access health facility<br>4 = Visiting family or friends<br>5 = Work related reasons<br>6 = Other (specify)<br>8 = Don't know<br>9 = Prefer not to answer
Q1.12	What is the main material of your home's exterior walls?	1 = No walls<br>2 = Cane / palm tree / trunks<br>3 = Earth<br>4 = Bamboo with mud<br>5 = Stone with mud<br>6 = Uncovered adobe / bamboo / wood with mud<br>7 = Reused wood<br>8 = Wood<br>9 = Cement<br>10= Stone with lime / cement<br>11= Bricks<br>12= Cement blocks<br>13= Coated adobe<br>14= Wood planks / shingles<br>15= Other, please describe: _______________

Q1.13	In your household, what type of fuel is primarily used for cooking?	1 =Electricity 2 = Biogas<br>3 = Kerosene<br>4 = Coal, ignite<br>5 = Charcoal<br>6 = Wood<br>7 = Straw / shrubs / grass<br>8 = Agricultural crops<br>9 = No food cooked in the house<br>10= Other, please describe: _______________
Q1.14	Does your household have at least one bed?	1 = Yes<br>2 = No<br>3 = Don't know<br>4 = No response
Q1.15	Does your household have at least one radio?	1 = Yes<br>2 = No<br>3 = Don't know<br>4 = No response
Q1.16	Over the last 12 months, what is your occupation, that is, what kind of work do you mainly do	1 = Not currently working<br>2 = Professional, technical, or managerial worker (salaried)<br>3 = Clerical worker<br>4 = Sales worker<br>5 = Self-employed agricultural worker<br>6 = Agricultural employee<br>7 = Household, domestic, or service worker<br>8 = Skilled manual worker<br>9 = Unskilled manual worker<br>10= Armed forces<br>11= Other, please describe: _______________<br>12= Don't know<br>13= No response

Section 2: Network Method, Household and Neighbor ties

In the following section, we want to know about the number of people you know who are your neighbors or live in your household.

Please think about all the people with your own household. By household, we mean people in most days of the previous week:

- Lived together under the same roof or in the same compound.
- Shared food from the same cooking pot

S/No	Question	Choices
Q2.1	Number of boys < 5 years of age?	Integer (total number)
Q2.2	Number of girls < 5 years of age?	Integer (total number)
Q2.3	Number of boys 5 - 18 years of age?	Integer (total number)
Q2.4	Number of girls 5 - 18 years of age?	Integer (total number)
Q2.5	Number of men 18+ years of age?	Integer (total number)
Q2.6	Number of women 18+ years of age?	Integer (total number)

Q2.7	How many people in your household have died since {recall_event}?	Integer (total number)
Please think of the 5 households closest to your household by walking distance. Please only tell me about the people who usually live in this household. By household, we mean people in most days of the previous week:<br><br>• Lived together under the same roof or in the same compound<br>• Shared food from the same cooking pot		
**Repeat following questions each of the closest 5 closest households by distance, closest household to furthest.**		
Q2.8	Number of boys < 5 years of age?	Integer (total number)
Q2.9	Number of girls < 5 years of age?	Integer (total number)
Q2.10	Number of boys 5 - 18 years of age?	Integer (total number)
Q2.11	Number of girls 5 - 18 years of age?	Integer (total number)
Q2.12	Number of men 18+ years of age?	Integer (total number)
Q2.13	Number of women 18+ years of age?	Integer (total number)
Q2.14	How many people in have died in {Neighbor household Num} since January 1st, 2023?	Integer (total number)
**Ask the following questions about the respondent's household and 5 closest neighbors combined**		
Q2.15	In your household, and your closest 5 neighbours, how many people have **LEFT** their localite or quartier since January 1st, 2023?	
Q2.16	How many births do you know of in your household, and the households of your 5 closest neighbors since January 1st, 2023?	
Q2.17	How many children under-5 years do you know in **your household, and the households of your 5 closest neighbours**, who had **MEASLES** since January 1st, 2023?	

Section 3: Network Method, Extended Kin

**We want to know about people you know who:**<br><br>• Reside in the same Zone De Sante as you<br>• You are blood related to<br>• Are still alive today		
Q3.1	How many of *YOUR OWN FEMALE CHILDREN** in {zone_de_sante_name} are:<br><br>• < 5 years of age<br>• 5–18 years of age<br>• 18+	Integer

Q3.2	How many of *YOUR OWN MALE CHILDREN** in {zone_de_sante_name} are:<br><br>• < 5 years of age<br>• 5–18 years of age<br>• 18+ years of age	Integer
Q3.3	How many of *YOUR OWN FEMALE GRANDCHILDREN** in {zone_de_sante_name} are:<br><br>• < 5 years of age<br>• 5–18 years of age<br>• 18+ years of age	Integer
Q3.4	How many of *YOUR OWN MALE GRANDCHILDREN ** in {zone_de_sante_name} are:<br><br>• < 5 years of age<br>• 5–18 years of age<br>• 18+ years of age	Integer
Q3.5	How many of *YOUR OWN SISTERS** in {zone_de_sante_name} are:<br><br>• < 5 years of age<br>• 5–18 years of age<br>• 18+ years of age	Integer
Q3.6	How many of *YOUR OWN BROTHERS** in {zone_de_sante_name} are:<br><br>• < 5 years of age<br>• 5–18 years of age<br>• 18+ years of age	Integer
Q3.7	How many of *YOUR OWN FEMALE COUSINS** in {zone_de_sante_name} are:<br><br>• < 5 years of age<br>• 5–18 years of age<br>• 18+ years of age	Integer
Q3.8	How many of *YOUR OWN MALE COUSINS** in {zone_de_sante_name} are:<br><br>• < 5 years of age<br>• 5–18 years of age<br>• 18+ years of age	Integer
Q3.9	How many of *YOUR OWN PARENTS** in {zone_de_sante_name} are:<br><br>• < 5 years of age<br>• 5–18 years of age<br>• 18+ years of age	Integer

Q3.10	How many of *YOUR OWN AUNTS** in {zone_de_sante_name} are:<br><br>• < 5 years of age<br>• 5–18 years of age<br>• 18+ years of age	Integer
Q3.11	How many of *YOUR OWN UNCLES** in {zone_de_sante_name} are:<br><br>• < 5 years of age<br>• 5–18 years of age<br>• 18+ years of age	Integer
Q3.12	How many of *YOUR OWN MALE GRANDPARENTS** in {zone_de_sante_name} are:<br><br>• < 5 years of age<br>• 5–18 years of age<br>• 18+ years of age	Integer
Q3.13	How many of *YOUR OWN FEMALE GRANDPARENTS** in {zone_de_sante_name} are:<br><br>• < 5 years of age<br>• 5–18 years of age<br>• 18+ years of age	
Ask below questions about all above kin relationships pooled		
Q3.14	Within your **EXTENDED FAMILY** which you counted, how many people do you know that have left their localite or quartier since January 1st, 2023 ?	
Q3.15	Within your **EXTENDED FAMILY** which you counted, how many people do you know that have **JOINED** their localite or quartier since January 1st, 2023?	
Q3.16	Within your **EXTENDED FAMILY**, how many births do you know since January 1st, 2023 within your extended family ?	
Q3.17	Within your **EXTENDED FAMILY**, how many older children (5+ years) or adults do you know who had **serious acute watery diarrhoea** since January 1st, 2023 ?	
Q3.18	Within your **EXTENDED FAMILY**, how many children under-5 years of age do you know who had **MEASLES** since January 1st, 2023?	

Section 4: Births

You reported:

- {num_births_neighbours} births from your household and your 5 closest neighbours
- {num_births_kin} births from your extended family

Q4.1	How many total, unique births really happened since January 1st, 2023	Integer
Repeat below questions for each birth reported		
Q4.2	What is your relationship to child #{birth_pos}?	

Q4.3	What is the family relationship?	
Q4.4	Do you know the sex of the child?	1 = Male<br>2 = Female;
Q4.5	Do you know the day, month, and year of child #{birth_pos} birth?	1 = Yes<br>2 = No<br>8 = Don't know<br>9 = Prefer not to answer
Q4.6	What is the **date of birth** for the child?	Date
Q4.7	If not exact date, can you estimate the **month-year of birth** for the child?	Month-Year
Q4.8	What was the outcome of this birth?	1 = Born, and alive<br>2 = Born, but now dead<br>3 = Child not born alive<br>4 = Don't Know<br>5 = Other, please describe: _____
**Section 5: Suspect Cholera**		
You reported:<br>• *{num_awd_neighbours} serious acute watery diarrhoea cases from your household and your 5 closest neighbours*<br>• *{num_awd_kin} serious acute watery diarrhoea cases from your extended family*		
Q5.1	*How many total, unique cases of serious acute watery diarrhoea really happened since January 1st, 2023?*	Integer
Repeat below questions for each unique case reported		
Q5.2	What was the sex of the person ?	1 = Male<br>2 = Female
Q5.3	What was the age in years of the person?	Integer
Q5.4	Did you observe the person directly when they were sick?	1 = Yes<br>2 = No<br>8 = Don't know<br>9 = Prefer not to answer
Q5.5	Did the person have at least 3 loose stools during a 24hour period?	1 = Yes<br>2 = No<br>8 = Don't know<br>9 = Prefer not to answer
Q5.6	Did the person have any vomiting?	1 = Yes<br>2 = No<br>8 = Don't know<br>9 = Prefer not to answer
Q5.7	Did the person have sunken eyes?	1 = Yes<br>2 = No<br>8 = Don't know<br>9 = Prefer not to answer
Q5.8	Do you know the **day, month, and year** that the person last had symptoms?	1 = Yes<br>2 = No<br>8 = Don't know<br>9 = Prefer not to answer
Q5.9	What is the **last date** that you are aware the person had symptoms?	Date
Q5.10	If not exact date, can you estimate the **month-year** that the person had symptoms?	Month-Year
Q5.11	Did the person seek health care?	1 = Yes<br>2 = No<br>8 = Don't know<br>9 = Prefer not to answer

Q5.12	If so, what place was health care sought?	1 = Govt. hospital<br>2 = Govt. health center<br>3 = Govt. health post<br>4 = Other govt. medical facility<br>5 = Private hospital<br>6 = Private clinic<br>7 = Other private facility<br>8 = NGO hospital<br>9 = NGO clinic<br>10= Other NGO facility<br>11= Other (please specify)<br>12= Don't know
Q5.13	What was the outcome of the person's illness?	1 = Person recovered<br>2 = Person still sick<br>3 = Person died<br>4 = Don't know<br>5 = Other (please specify)
**Section 6: Suspect Measles**		
**You reported:**<br>• {num_measles_neighbours} measles cases from your household and your 5 closest neighbours<br>• {num_measles_kin} measles cases from your extended family		
Q6.1	How many children (under-5 years) do you know who had measles since January 1st, 2023?	Integer
Repeat below questions for each person reported		
Q6.2	What was the sex of the child ?	1 = Male<br>2 = Female
Q6.3	What was the age in years of the child?	Integer
Q6.4	Did you observe the child directly when they were sick?	1 = Yes<br>2 = No<br>8 = Don't know<br>9 = Prefer not to answer
Q6.5	Did the child have a rash on their head and/or neck?	1 = Yes<br>2 = No<br>8 = Don't know<br>9 = Prefer not to answer
Q6.6	Did the child have fever?	1 = Yes<br>2 = No<br>8 = Don't know<br>9 = Prefer not to answer
Q6.7	Do you know the **day, month, and year** that the child had measles?	1 = Yes<br>2 = No<br>8 = Don't know<br>9 = Prefer not to answer
Q6.8	What is the **last date** that you are aware the child had measles symptoms?	Date
Q6.9	If not exact date, can you estimate the **month-year** that the child had measles symptoms?	Month-Year
Q6.10	Did the child seek health care?	1 = Yes<br>2 = No<br>8 = Don't know<br>9 = Prefer not to answer

Q6.11	If so, what place was health care sought?	1 = Govt. hospital<br>2 = Govt. health center<br>3 = Govt. health post<br>4 = Other govt. medical facility<br>5 = Private hospital<br>6 = Private clinic<br>7 = Other private facility<br>8 = NGO hospital<br>9 = NGO clinic<br>10= Other NGO facility<br>11= Other (please specify)<br>12= Don't know
Q6.12	What was the outcome of the child's illness?	1 = Person recovered<br>2 = Person still sick<br>3 = Person died<br>4 = Don't know<br>5 = Other (please specify)

Section 7: Deaths

You reported:

- {num_deaths_hh} deaths from your own household
- {num_deaths_neighbours} deaths from your 5 closest neighbours
- {num_deaths_kin} deaths from your extended family

Q7.1	How many total, unique deaths really happened since {recall_event}?	Integer
Repeat below questions for each person reported		
Q7.2	What was the first name of the deceased individual?	Text
Q7.3	What was the family name of the deceased individual?	Text
Q7.4	Was [name_deceased] known by any other names or nicknames?	Text
Q7.5	What was the sex of [name_deceased]?	1 = Male<br>2 = Female;

Q7.6	What was the age in completed years of [name_deceased] ?	Integer
Q7.7	Do you know the day, month, and year of [name_deceased] birth?	1 = Yes<br>2 = No<br>8 = Don't know<br>9 = Prefer not to answer
Q7.8	What is the **date of birth** for [name_deceased]?	Date
Q7.9	If not exact date, can you estimate the **month-year of birth** for [name_deceased] ?	Month-Year
Q7.10	Do you know the day, month, and year that [name_deceased] passed away?	1 = Yes<br>2 = No<br>8 = Don't know<br>9 = Prefer not to answer
Q7.11	Do you know the exact date that [name_deceased] passed away?	Date
Q7.12	If not, please estimate the month-year of death as close as possible?	Month-Year
Q7.13	What was the main cause of death for [name_deceased]?	1 = Acute disease<br>2 = Chronic disease<br>3 = Intentional violence<br>4 = Accident/trauma<br>5 = Post-partum (0-42 days)<br>6 = During pregnancy<br>7 = During delivery<br>8 = Other (please specify)

		9 = Don't know
Q7.14	Where did the [name_deceased] pass away?	1 = Current location of residence<br>2 = Health facility at current location of residence<br>3 = During migration or displacement<br>4 = At last place of residence<br>5 = Health facility at last place of residence
Q7.15	Did [name_deceased] seek health care in the 2 weeks before dying?	1 = Yes<br>2 = No<br>8 = Don't know<br>9 = Prefer not to answer
Q7.16	If so, what place was health care sought?	1 = Govt. hospital<br>2 = Govt. health center<br>3 = Govt. health post<br>4 = Other govt. medical facility<br>5 = Private hospital<br>6 = Private clinic<br>7 = Other private facility<br>8 = NGO hospital<br>9 = NGO clinic<br>10= Other NGO facility<br>11= Other (please specify)<br>12= Don't know
Q7.17	If not, what was the main reason for not seeking care in a health structure/facility?	1 = Immediate death<br>2 = No money/consultation too expensive<br>3 = Too sick to seek care<br>4 = Not sick enough to seek care<br>5 = Health facility too far away<br>6 = Went to a traditional healer<br>7 = No time to go/too busy to go<br>8 = No trust in the health facility<br>9 = Safety issue<br>10= Care was refused at the health center<br>11= Other please specify<br>12= Don't know
Q7.18	In your own words, can you provide any other details about the circumstances of [name_deceased]'s death?	[Text description]
Q7.19	Was [name_deceased] a part of your own household?	1 = Yes<br>2 = No<br>8 = Don't know<br>9 = Prefer not to answer
Q7.20	Was [name_deceased] a membre of the community you currently live in?	1 = Yes<br>2 = No<br>8 = Don't know<br>9 = Prefer not to answer
Q7.21	If no, what Zone de Sante did [name_deceased] live at the time of death?	[Select one – contextual list]
Q7.22	If no, what Aire de Sante did [name_deceased] live at the time of death?	[Select one – contextual list]
Q7.23	If no, what Village did [name_deceased] live at the time of death?	[Select one – contextual list]

Q7.24	We would like to follow up more closely with the household of [name_deceased] to better understand the causes of their death. This will help us understand the causes of high mortality in Tanganyika Province so the health department and NGOs can better plan their response.<br><br>We would like to ask your permission to follow up with [name_deceased]'s household directly to better understand the causes of death. We would not disclose your information that you told us about the death, but it would increase the risk of breaching your confidentiality if we discussed with the household. If you are not comfortable with us following up with the household, please tell us. We will only follow up with them if you give us permission to do so.<br><br>Do we have your permission to follow up with the household of [name_deceased]?	1 = Yes<br>2 = No<br>8 = Don't know<br>9 = Prefer not to answer
Q7.25	Do you have any phone number you can share for [name_deceased]'s household?	1 = Yes<br>2 = No<br>8 = Don't know<br>9 = Prefer not to answer
Q7.26	Do we have your permission to follow up with [name_deceased] household with some questions about cause of death?	1 = Yes<br>2 = No<br>8 = Don't know<br>9 = Prefer not to answer
Q7.27	Phone number	Phone Number
Q7.28	Is there anyone else we could call by phone who could connect us with [name_deceased]'s household?	1 = Yes<br>2 = No<br>8 = Don't know<br>9 = Prefer not to answer
Q7.29	Phone number (alternate):	[Phone Number]
Q7.30	Do you have any other information on how we could reach or contact [name_deceased]'s household?	[Text Description]

References

[1] Dennis M. Feehan, Mary Mahy, and Matthew J. Salganik. The Network Survival Method for Estimating Adult Mortality: Evidence From a Survey Experiment in Rwanda. *Demography*, 54(4):1503–1528, August 2017. ISSN 0070-3370, 1533-7790. doi: 10.1007/s13524-017-0594-y. URL https://read.dukeupress.edu/demography/article/54/4/1503/167730/The-Network-Survival-Method-for-Estimating-Adult.

[2] Francesco Checchi and Les Roberts. Interpreting and using mortality data in humanitarian emergencies: A primer for non-epidemiologists. Technical report, 2005. URL https://odihpn.org/publication/interpreting-and-using-mortality-data-in-humanitarian-emergencies/.

[3] Christopher McCarty, Peter D. Killworth, H. Russell Bernard, Eugene C. Johnsen, and Gene A. Shelley. Comparing Two Methods for Estimating Network Size. *Human Organization*, 60(1):28–39, 2001. ISSN 0018-7259. URL https://www.jstor.org/stable/44126693.

[4] H Russell Bernard, Tim Hallett, Alexandrina Iovita, Eugene C Johnsen, Rob Lyerla, Christopher McCarty, Mary Mahy, Matthew J Salganik, Tetiana Saliuk, Otilia Scutelniciuc, Gene A Shelley, Petchsri Sirinirund, Sharon Weir, and Donna F Stroup. Counting hard-to-count populations: The network scale-up method for public health. *Sexually Transmitted Infections*, 86(Suppl_2):ii11–ii15, December 2010. ISSN 1368-4973. doi: 10.1136/sti.2010.044446. URL https://www.ncbi.nlm.nih.gov/pmc/articles/PMC3010902/.

[5] Holly E. Reed and Charles B. Keely. Understanding Mortality Patterns in Complex Humanitarian Emergencies. In *Forced Migration & Mortality*. National Academies Press (US), 2001. URL https://www.ncbi.nlm.nih.gov/books/NBK223340/.

[6] Ian M. Timæus. Measurement of Adult Mortality in Less Developed Countries: A Comparative Review. *Population Index*, 57(4):552–568, 1991. ISSN 0032-4701. doi: 10.2307/3644262. URL https://www.jstor.org/stable/3644262.

[7] Bruno Lankoandé, Bruno Masquelier, Pascal Zabre, Hélène Bangré, Géraldine Duthé, Abdramane B. Soura, Gilles Pison, and Sié Ali. Estimating mortality from census data: A record-linkage study of the Nouna Health and Demographic Surveillance System in Burkina Faso. *Demographic Research*, 46:653–680, April 2022. ISSN 1435-9871. doi: 10.4054/DemRes.2022.46.22. URL https://www.demographic-research.org/articles/volume/46/22.

[8] Kenneth Hill, Peter Johnson, Kavita Singh, Anthony Amuzu-Pharin, and Yagya Kharki. Using census data to measure maternal mortality: A review of recent experience. *Demographic research*, 39:337–364, 2018. ISSN 1435-9871. doi: 10.4054/DemRes.2018.39.11. URL https://www.ncbi.nlm.nih.gov/pmc/articles/PMC6903798/.

[9] Prudence Jarrett, Frank J. Zadravecz, Jennifer O'Keefe, Marius Nshombo, Augustin Karume, and Les Roberts. Evaluation of a population mobility, mortality, and birth

surveillance system in South Kivu, Democratic Republic of the Congo. *Disasters*, 44 (2):390–407, April 2020. ISSN 1467-7717. doi: 10.1111/disa.12370.

[10] Michael R. Elliott and Richard Valliant. Inference for Nonprobability Samples. *Statistical Science*, 32(2):249–264, 2017. ISSN 0883-4237. URL https://www.jstor.org/stable/26408228.

[11] Maksym Bondarenko, David Kerr, Alessandro Sorichetta, Andrew Tatem, and WorldPop,. Census/projection-disaggregated gridded population datasets for 51 countries across sub-Saharan Africa in 2020 using building footprints., 2020. URL https://www.worldpop.org/doi/10.5258/SOTON/WP00682.

[12] Dennis M. Feehan, Aline Umubyeyi, Mary Mahy, Wolfgang Hladik, and Matthew J. Salganik. Quantity Versus Quality: A Survey Experiment to Improve the Network Scale-up Method. *American Journal of Epidemiology*, 183(8):747–757, April 2016. ISSN 0002-9262. doi: 10.1093/aje/kwv287. URL https://doi.org/10.1093/aje/kwv287.

[13] Smart Survey. Enquete Nutritionnelle Smart Territoire De Kalemie. Technical report, studfee, 2022.
